# Invasion of Human Retinal Pigment Epithelial Cells by *Porphyromonas gingivalis* leading to Vacuolar/Cytosolic localization and Autophagy dysfunction *In-Vitro*

**DOI:** 10.1038/s41598-020-64449-8

**Published:** 2020-05-04

**Authors:** Pachiappan Arjunan, Radhika Swaminathan, Jessie Yuan, Mohamed Al-Shabrawey, Diego G. Espinosa-Heidmann, Julian Nussbaum, Pamela M. Martin, Christopher W. Cutler

**Affiliations:** 1 Department of Periodontics, Dental College of Georgia, Augusta University, Augusta, USA; 2 Department of Oral Biology and Diagnostic Sciences, Augusta University, Augusta, USA; 3 Department of Ophthalmology, Augusta University, Augusta, USA; 4 Department of Biochemistry and Molecular Biology, Augusta University, Augusta, GA 30912 USA

**Keywords:** Cell biology, Immunology, Microbiology, Diseases, Pathogenesis

## Abstract

Recent epidemiological  studies link Periodontal disease(PD) to age-related macular degeneration (AMD). We documented earlier that *Porphyromonas gingivalis(Pg)*, keystone oral-pathobiont, causative of PD, efficiently invades human gingival epithelial and blood-dendritic cells. Here, we investigated the ability of dysbiotic *Pg*-strains to invade human-retinal pigment epithelial cells(ARPE-19), their survival, intracellular localization, and the pathological effects, as dysfunction of RPEs leads to AMD. We show that live, but not heat-killed *Pg-*strains adhere to and invade ARPEs. This involves early adhesion to ARPE cell membrane, internalization and localization of *Pg* within single-membrane vacuoles or cytosol, with some nuclear localization apparent. No degradation of *Pg* or localization inside double-membrane autophagosomes was evident, with dividing *Pg* suggesting a metabolically active state during invasion. We found significant downregulation of autophagy-related genes particularly, autophagosome complex. Antibiotic protection-based recovery assay further confirmed distinct processes of adhesion, invasion and amplification of *Pg* within ARPE cells. This is the first study to demonstrate invasion of human-RPEs, begin to characterize intracellular localization and survival of *Pg* within these cells. Collectively, invasion of RPE by *Pg* and its prolonged survival by autophagy evasion within these cells suggest a strong rationale for studying the link between oral infection and AMD pathogenesis in individuals with periodontitis.

## Introduction

Periodontal disease (PD or periodontitis) is a highly prevalent (50%)^1^ inflammatory oral disease in the US. PD destroys the supportive connective tissue of teeth and alveolar bone and thereby is a primary cause of tooth loss. Over the years, studies have proven that chronic inflammation instigated in the oral cavity has implications for initiation and/or progression of many systemic diseases^2–11^. Markedly, type-2 diabetes^3^, cardiovascular disease^12^, stroke^7^, respiratory infections like pneumonia^5^, pre-term birth^9^, Alzheimer’s disease^6,10^, Parkinson’s disease^13^, liver disease^14^, rheumatoid arthritis^8^, intra- and extra-oral cancers^15^ are linked to periodontitis.

*P. gingivalis*^16,17^, an effective late colonizer of oral tissues and a red complex bacterium is highly associated with PD. This gram-negative anaerobe expresses gingipain proteinase (kgp/rgp), fimbriae (major and minor), lipopolysaccharides (LPS), collagenase, and endotoxin (TLR2/TLR4 agonist/antagonist), all of which contribute to its virulence. The pathogenicity of *P. gingivalis*, including its ability to colonize tooth surfaces, moderate host cytokine responses, cause tissue damage and inhibit cell apoptosis^18^ have been studied. Recent studies emphasize the ability of *P. gingivalis* to hijack host autophagy pathways to establish a successful replicative niche for extended survival in gingival epithelial cells (GECs)^19^. The major *FimA* and minor *Mfa1* fimbriae facilitate invasion of host epithelial cells, endothelial cells, and dendritic cells (DCs) by *P. gingivalis*, enabling it to avoid the host defense mechanism^20^ and in the case of DCs, disseminate throughout the body. The tissue destruction caused by PD is not directly by the bacterial byproducts but by the cascade of host inflammatory response. Therefore, the “mobile microbiome” is capable of inhabiting extra-oral sites culminating in distant infections and persistent general inflammation^21^.

Age-related Macular Degeneration (AMD) is a highly prevalent eye disease that imposes significant impact on public health, causing irreversible loss of central vision in the elderly^22,23^. AMD occurs through a combined non-neovascular and neovascular derangement^24^. The hallmark of dry or nonexudative form of AMD is the deposition of extracellular material and drusen formation underneath the retinal pigment epithelial (RPE) layer, leading to loss of retinal photoreceptors^25^. Alternately, the exudative or wet type is characterized by choroidal neovascularization underneath the RPE. There are strong evidences stipulating that inflammation plays a major role in AMD^26^. Many studies suggest that drusen formation may initiate an inflammatory cascade that directs AMD progression^25^. It is reported that sections of drusen contain increased levels of autophagic markers^27^. Elevated oxidative stress and dysregulated autophagy induce RPE degeneration and subsequently chronic inflammation in the retina^25,28^ which is pathognomonic of AMD. The sequelae involves RPE degeneration ensuing in disrupted Bruch’s membrane, increased vascular permeability and retinal neovascularization, endangering central vision^26^. A complex etiology and unknown triggers limit a clear understanding of AMD pathophysiology and effective cure.

Chronic oral inflammation in PD is sustained by dysbiosis of the “proximal gut”, i.e. the oral cavity, and a leaky attachment apparatus. Recent epidemiological evidence revealed a 2-fold higher risk of AMD in PD patients with alveolar bone loss^11,29^ although the mechanisms are unidentified. Pathologic shifts in oral microbiota is also linked to higher mortality rates^30^. Specific microbiota of human nasopharynx is linked to AMD^31^. Despite these observations, a limited understanding exists about the role of dysbiotic oral pathobionts in AMD pathology indicating a critical knowledge gap and an imperative need to probe the underlying molecular mechanisms. Moreover, the dissemination of oral microbes to the eye structure and their interactions that could contribute to the development of AMD have not been investigated yet. This may represent a novel opportunity to understand the mechanisms underlying the pathological association of AMD in coexisting chronic oral inflammation.

The anatomical, cellular and molecular specializations of the eye are thought to maintain an immune-privileged state^32^ as the entry of immune cells to this organ was thought to be nonexistent^33^. However, recent studies show recruitment of immune cells to the eye following retinal injury through infections or inflammation. The low-grade inflammation, sustained by dysbiosis and a leaky gut^34^, has been identified to contribute to the development of AMD.

Previously, we observed mechanisms for how *P. gingivalis* and its fimbrial-mutant strains invade and survive in human DCs^35^, however, the ability of *P. gingivalis* or other oral microbes to invade RPE have not been demonstrated. The RPE is a highly specialized, metabolically active layer which continuously recycles the shed photoreceptor cells and processes the metabolic wastes by autophagy and support the visual function^36,37^. Moreover, an intact blood retinal barrier (BRB) is pivotal to maintain a homeostatic retinal microenvironment. The BRB consists of dual layer with inner (tight junctions between retinal capillary endothelial cells) and the outer (tight junctions between RPE cells) compartments. Breakdown of the inner endothelial BRB is reported in diabetic retinopathy and that of outer BRB in case of AMD^38^.

Therefore, our goal is to examine the hypothesis that the dysbiotic oral pathogen *P. gingivalis* and its isogenic mutants, at different multiplicities of infection, are capable of invading human RPE cells (ARPE-19) *in-vitro* and surviving within as an intracellular pathogen. Using a combination of immunofluorescence, SEM, TEM, confocal microscopy, qPCR, antibiotic protection and survival assay, we show that *P. gingivalis* adheres to and invades RPEs, with the latter being an active process, requiring that the invading strain be viable and express fimbriae to evade autophagy, as an intracellular pathogen of RPEs. So, this will be the first study to demonstrate the invasion and internalization of the oral pathogen *Pg* and its mutant strains in RPE cells *in-vitro*. These emanating findings will provide a platform to get a new insight into the pathogenic role of *P. gingivalis*, comprehend the underlying mechanisms and plausibly expand the rudimentary knowledge of the association of AMD and PD.

## Results

### **Invasion by***P. gingivalis***(*Pg*381) of Human Retinal Pigment Epithelial (ARPE-19) cells**

**Figure 1:**
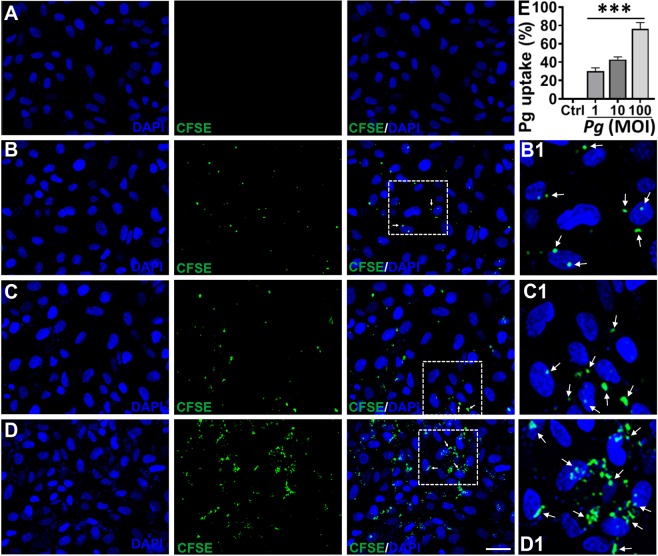
Uptake of *P. gingivalis* by Human Retinal Pigment Epithelial (ARPE-19) cells. (**A–D**) Confocal images of human retinal pigment epithelial cells infected with CFSE-pre-labeled *P. gingivalis* (*Pg*381) after 24 hours at 1 (**B**), 10 (**C**) and 100 (**D**) MOI compared with uninfected control (**A**) group. The results show the apparent uptake of *Pg*381 by ARPE-19 cells at 1, 10, and 100 MOI as detected by confocal microscopy (white arrows). Boxed areas in **B, C** and **D** show an enlarged region as B1, C1 and D1, respectively. (Green-CFSE; Blue-DAPI). Scale bar: 20 µm. Images are representative of three independent experiments from three different passages in a double-blind manner. (**E**) The quantification analysis shows the uptake of *Pg* relative to the uninfected control and plotted as percentage. Analysis of fluorescent levels using IMAGEJ software revealed significant uptake of CFSE labeled *P. gingivalis* in all 1, 10 and 100 MOI groups compared with control group. The intensity of CFSE-labeled *Pg* were measured from six randomly selected images from three independent experiments. The data shown represent the mean ± standard error of the mean of three experiments (n = 3). One-way ANOVA analysis was used to compare the means of intensity of different groups/concentrations and Tukey’s multiple comparisons test with three different experiments (n = 3). ***P < 0.001. MOI - Multiplicity of Infection.

To determine whether *P. gingivalis* invades human RPE, ARPE-19 cells were cocultured with CFSE-labeled *Pg*381 at 1, 10 and 100 multiplicity of infection (MOI) (Fig. 1B–D) and uptake quantified (Fig. 1E), compared with uninfected control (Fig. 1A) after 24 hours. Observed was substantial adhesion and uptake of *Pg*381 by ARPE cells in a dose (MOI)-dependent manner as detected by confocal imaging analysis (Fig. 1B–D). It was interesting to note that *Pg*381 is capable of invading ARPE cells even at 1 MOI (Fig. 1B), which can be clearly observed in the cytosol and around the cell nuclei. Quantitative analysis showed significant uptake of *Pg*381 by ARPE-19 cells at 1, 10 and 100 MOI (Fig. 1E). This data also demonstrates that there was considerable increase in the invasion efficiencies of *P. gingivalis* with increasing MOI.

### **Live but not heat killed***P. gingivalis***invade retinal epithelial cells**

**Figure 2:**
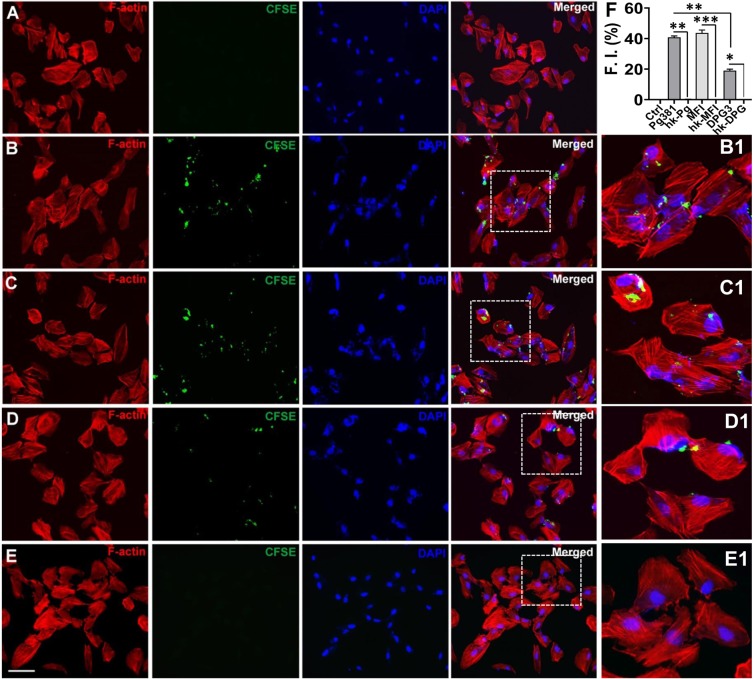
Live *P. gingivalis* and its isogenic mutant strains invades Human Retinal Pigment Epithelial (ARPE-19) cells. (**A–E**) ARPE-19 cells were co-cultured with live CFSE-labeled *Pg*381, MFI and DPG3 (10 MOI) or heat killed (HK) all fimbriated *Pg* strains for 24 hours and compared to uninfected control. After fixation and permeabilization, the infected ARPE cells were stained with rhodamine-phalloidin (F-actin for cell surface) and DAPI (nuclear stain) and then examined by confocal microscopy. Representative images show the live *Pg381* (**B**), MFI (**C**) and DPG3 (**D**) can enter ARPE-19 cells but not the heat-killed *Pg381* (**E**), HK-MFI and HK-DPG3 (refer Fig. S1A–C). Boxed areas in B, C, D and E show an enlarged region as B1, C1, D1 and E1, respectively. Red - F-actin; Green - CFSE; Blue - DAPI. The data shown are representative of three similar results. Scale bar: 20 µm. (**F**) The quantification analysis shows significant invasion of all fimbriated live *Pg* strains compared to the uninfected control as well as their respective heat-killed bacteria and plotted as percentage. The fluorescence intensity of CFSE-labeled *Pg* strains were measured as shown in Fig. 1. There were no significant differences between *Pg*381 and MFI groups. The analysis of the intensity used Kruskal-Wallis test of different groups and Dunn’s test for multiple comparisons 3 different experiments. *P < 0.05; **P < 0.01; ***P < 0.001. The error bars indicate ± SEM (n = 3). F.I. – Fluorescent Intensity.

Next, we tested if heat-killed *Pg*381 or the isogenic fimbriae mutant strains MFI and DPG3 can invade the retinal epithelial cells. ARPE cells infected with CFSE labeled live *Pg*381 and its mutant strains at 10 MOI for 24 hours showed bacterial invasion upon examination by confocal imaging (Fig. 2A–E). Invasion was confirmed by the presence of *P. gingivalis* within the ARPE cell boundary surrounded by actin filaments through several consecutive z-sections. In the ARPE cells infected with live *Pg*381, MFI and DPG3, many were clearly visible around the cell membrane and nuclei as well cytosol at 10 MOI (Fig. 2B–D) compared with uninfected control (Fig. 2A). Interestingly, DPG3 strain, which lacks the major or *FimA* fimbriae was less able to invade ARPE cells (Fig. 2D). However, none of the heat-killed *Pg*381 (Fig. 2E and S1A) and its mutant strains (Fig. S1B,C) invaded ARPE-19 cells or were membrane-bound. The quantification analysis demonstrated significant invasion of *Pg*381, MFI and DPG3 compared to their respective heat-killed bacteria and uninfected control as well (Fig. 2F). Percentage of invasion of *Pg*381 is comparable with MFI, both of which express the major *FimA*, but MFI lacks minor or *Mfa1* fimbriae, suggesting *Mfa1* is not required for invasion of ARPE as it is for DCs. These results suggest that only live *P. gingivalis* and its mutant strains can efficiently invade retinal epithelial cells and that the major *FimA* fimbriae is required for optimum invasion.

### **Primary interaction of***P. gingivalis***with human retinal epithelial cell membrane**

**Figure 3:**
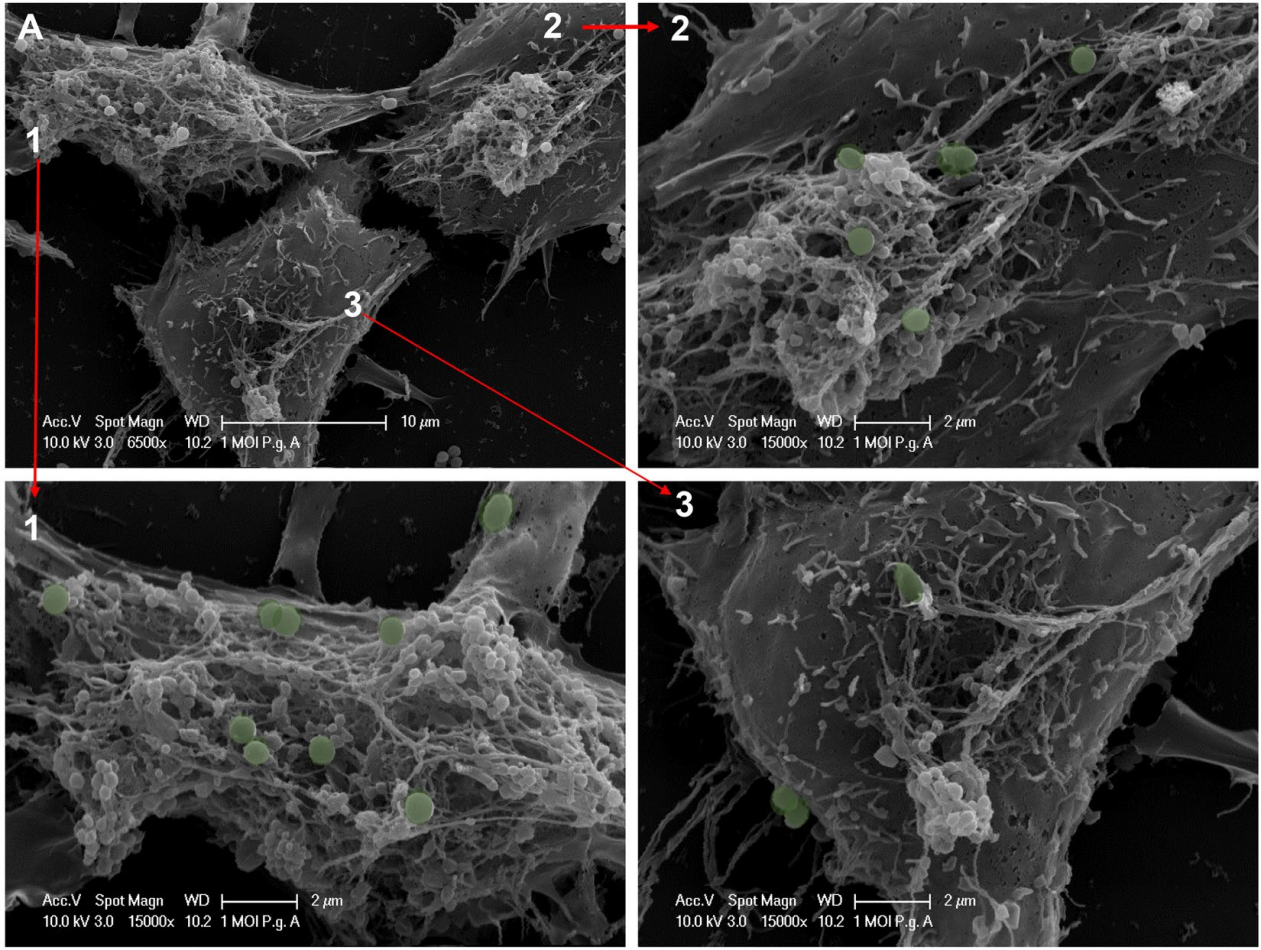
Primary interaction of *P. gingivalis* with ARPE cell membrane. (**A**) Adherence of *P. gingivalis* to ARPE cells examined by scanning electron microscopy (SEM). The sections show the primary interaction of *Pg*381 infected human retinal epithelial cells for 1 hour with 1 MOI. It clearly demonstrates apical and basolateral membrane engagement of *Pg*. Images A1, A2 and A3 (green stains for *Pg*381 are computer generated) shows the high magnification of 3 A. Scale bar: – A: 10 µm; 1, 2 and 3: - 2 µm. Refer fig. S2 for different images of SEM at 1 (Fig. 2A,B) and 10 (Fig. 2C,D) MOI of *Pg*381 for 24 hours infection.

To examine early interactions of *P. gingivalis* with ARPE membrane at 1-hour, fixed cells were examined by scanning electron microscopy (SEM) as described earlier^39^. SEMs show initial interaction of *Pg* with the outer membrane of ARPE after 1-hour of infection (Fig. 3 and S2). *Pg*381 was able to adhere and tightly engage the human ARPE cell surface at 1 hour at both 1 (Fig. 3A 1–3 and S2A,B) and 10 (Fig. S2C,D) MOI. In addition, we also observed *P. gingivalis* highly clustered and engaged with cell membrane at 10 MOI (Fig. S2C,D).

### **High interaction and invasion of***P. gingivalis***within ARPE-19 cells**

**Figure 4:**
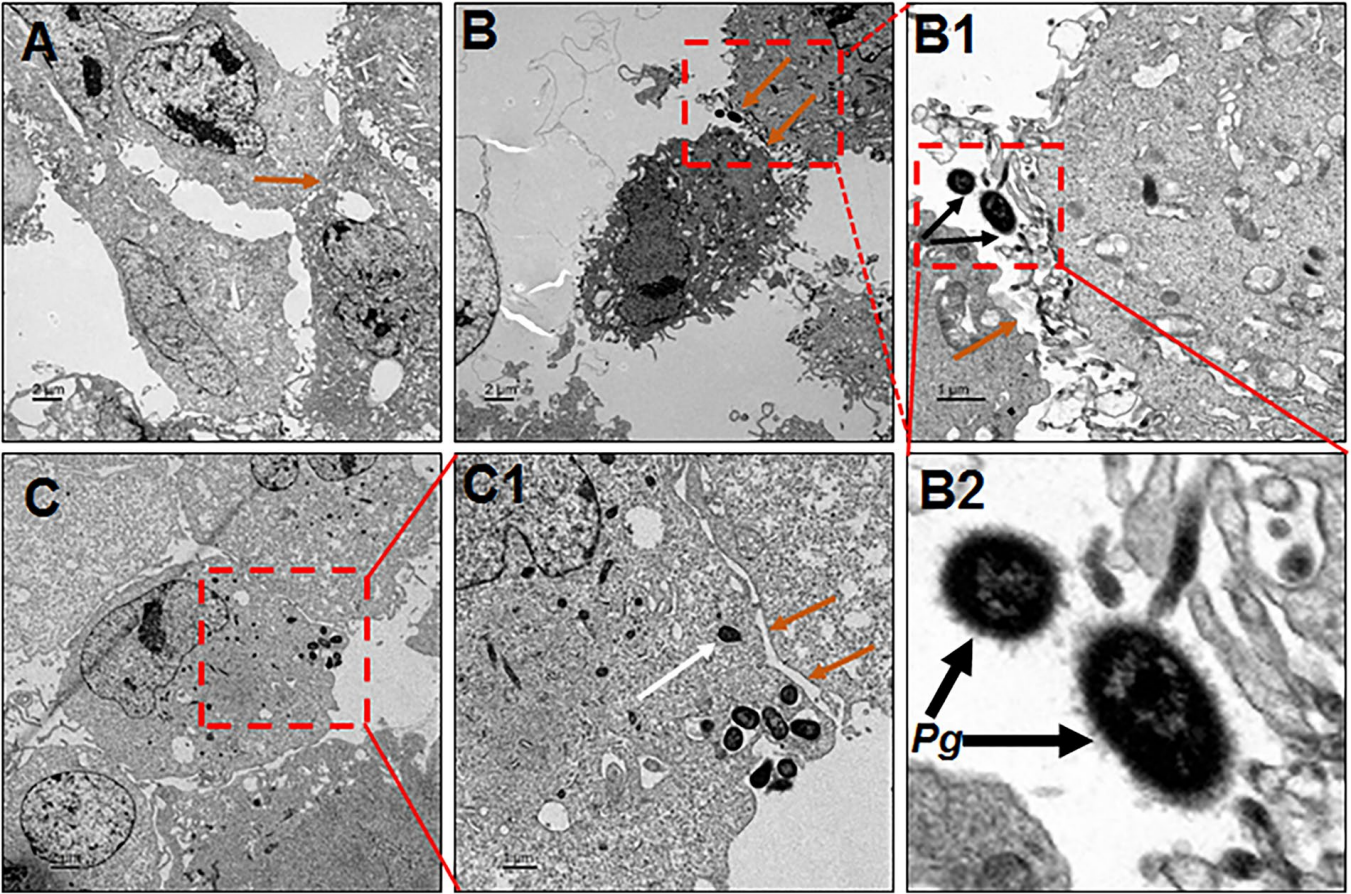
High interaction and invasion of *P. gingivalis* within ARPE cells. (**A–C**) Transmission electron microscopy (TEM) of human retinal pigment epithelial (ARPE) cells infected with *P. gingivalis* at 1 (B–B2) and 10 (C and C1) MOI for 1 hour compared with uninfected control (**A**). The sections show the clear host pathogen interaction and invasion of *Pg*381 (**B,C**) in ARPE cells relative to uninfected control (**A**). Boxed areas in B, B1 and C show an enlarged/magnified region B1, B2 and C1, respectively. (B1) *P. gingivalis* adhering to ARPE surface (black arrow). (B2) Enlarged section (of B1) showing the *Pg* fimbriae-mediated adhesion and their interaction with ARPE cells clearly. (**C**) *P. gingivalis* internalized and freely occupied the cytoplasm of ARPE cells (white arrow). Orange arrows indicate several epithelial junctions such long and short tight junctions between ARPEs, particularly, B, B1,C1- shows the disintegrated or altered tight junctions (orange arrows) and basolateral entry of *Pg*. Scale bars:- A-C: 2μm, B1, C1: 1μm), (n = 4). Note:- Refer Fig. S3 for another set of representative images.

At 1-hour after bacterial co-culture with ARPE cells, the ARPEs were imaged for interaction and intracellular *P. gingivalis* by transmission electron microscopy (TEM). TEM delineated the intimate surface interaction, and adhesion of *P. gingivalis*, leading to invasion of *Pg* in ARPE cells after exposure at 1 (Fig. 4B,B1,B2) and 10 MOI (Fig. 4C,C1 and S3C,C1) for 1-hour, relative to uninfected control (Fig. 4A). It’s interesting to note at high power the visible *Pg*381 fimbriae and their interaction with ARPE cells (Fig. 4B2), particularly aggregated invasion at 10 MOI (Fig. 4C-C1 and S3C,C1), which is consistent with results observed in SEM images (Fig. S2C,D). These results confirm that the fimbriated *Pg*381 was able to interact and invade the ARPE cells.

### *P. gingivalis***accesses single membrane vesicles, cytosol and nucleus of ARPE cells**

**Figure 5:**
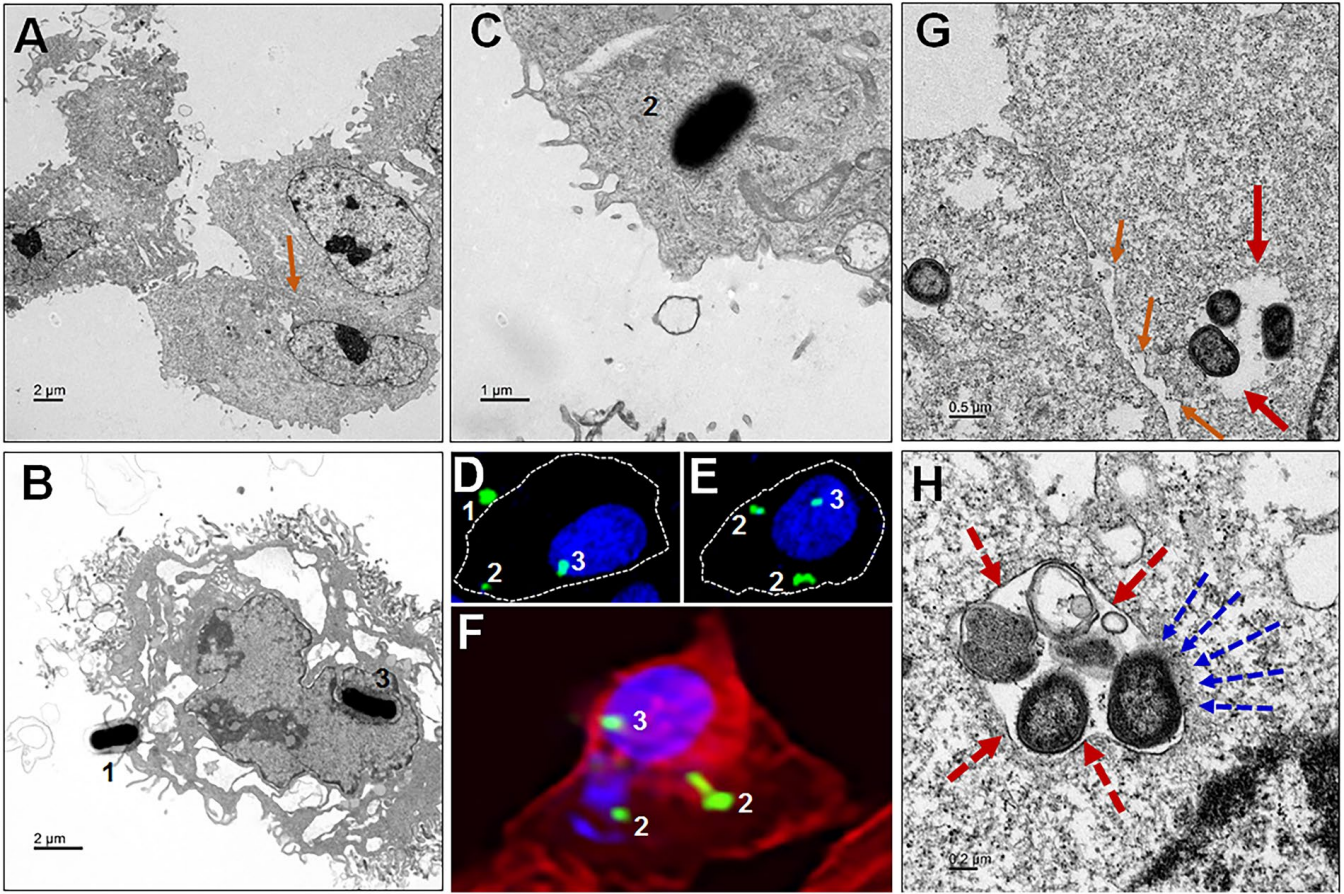
*P. gingivalis* manipulation of ARPE cell entry and escape to cytosol. (**A**) Uninfected control (orange arrow shows the tight junction). (**B**) *Pg* interact with ARPE cells (1) and reached the cell nucleus (3). (**C**) Invaded *Pg* freely occupy the cytoplasm of ARPE cells (2). (**D–F**) Confocal images shows the three stages (1. host-pathogen interaction to enter, 2. Invasion and 3, Internalization) and invasion location of the *Pg* into ARPE cells (membrane, cytoplasm and nucleus), which is consistent to the TEM as shown in (**B,C**) as well. (**D,E**) White dotted lines indicates the cell membrane boundary as visualized by F-actin (**F**). (**G,H**) The adhesive properties of fimbriae allow *P. gingivalis* to invade host cells and escape the host immune surveillance. (**G**) Bacteria in vacuole to evade the host immune defense and survive long (red arrows). Intracellular *Pg* is found within vesicular structures with single enclosed membranes (dotted red arrows); also noticed *P*g organisms inside the retinal epithelial cell, without endocytic vacuoles surrounding them, probably escaped from the vacuole, is evident (**H**). Also observed loss of integrity of tight junctions in between RPEs (**G**), compared to uninfected control (**A**) - orange arrows. Note:- the vacuole membrane breaking and *Pg* escape to the cytosol (dotted blue arrows). (Scale bar:- A-B: 2 µm, C: 1 µm, G: 0.5 µm, H: 0.2 µm; D, E and F: enlarged region from Figs. 1B and 2B, respectively). Refer the low magnification in Fig. S4 for the complete view.

We next performed TEM imaging analysis to assess the intracellular localization *P. gingivalis* within ARPE cells. Available evidence suggests that the intracellular environment can protect certain microbes until they develop a full complement of virulence factors^40^. Here it is shown that *Pg*381 enters single membrane vacuole structures, as well as freely occupying the cytoplasm and reaching the cell nucleus (Fig. 5B–H) compared to uninfected control (Fig. 5A). Confocal imaging analysis also demonstrates the three stages of entry from adhesion to invasion and intracellular localization to cytoplasm and nucleus (Fig. 5D–F), consistent with TEM results (Fig. 5B,C and S4B). The formation of single cell vesicles enclosing *P. gingivalis* was evident, as was lack of visible degradation (Fig. 5G,H and S4C,D). The presence of *P*g in the cytosol, without vesicular membrane surrounding them, suggests escape from the vacuole. Also evident is some disintegration of vacuole membrane and *Pg* re-localization to the cytosol (Fig. 5H).

### **High internalization and intracellular content of*Pg*strains within ARPE-19 cells**

**Figure 6:**
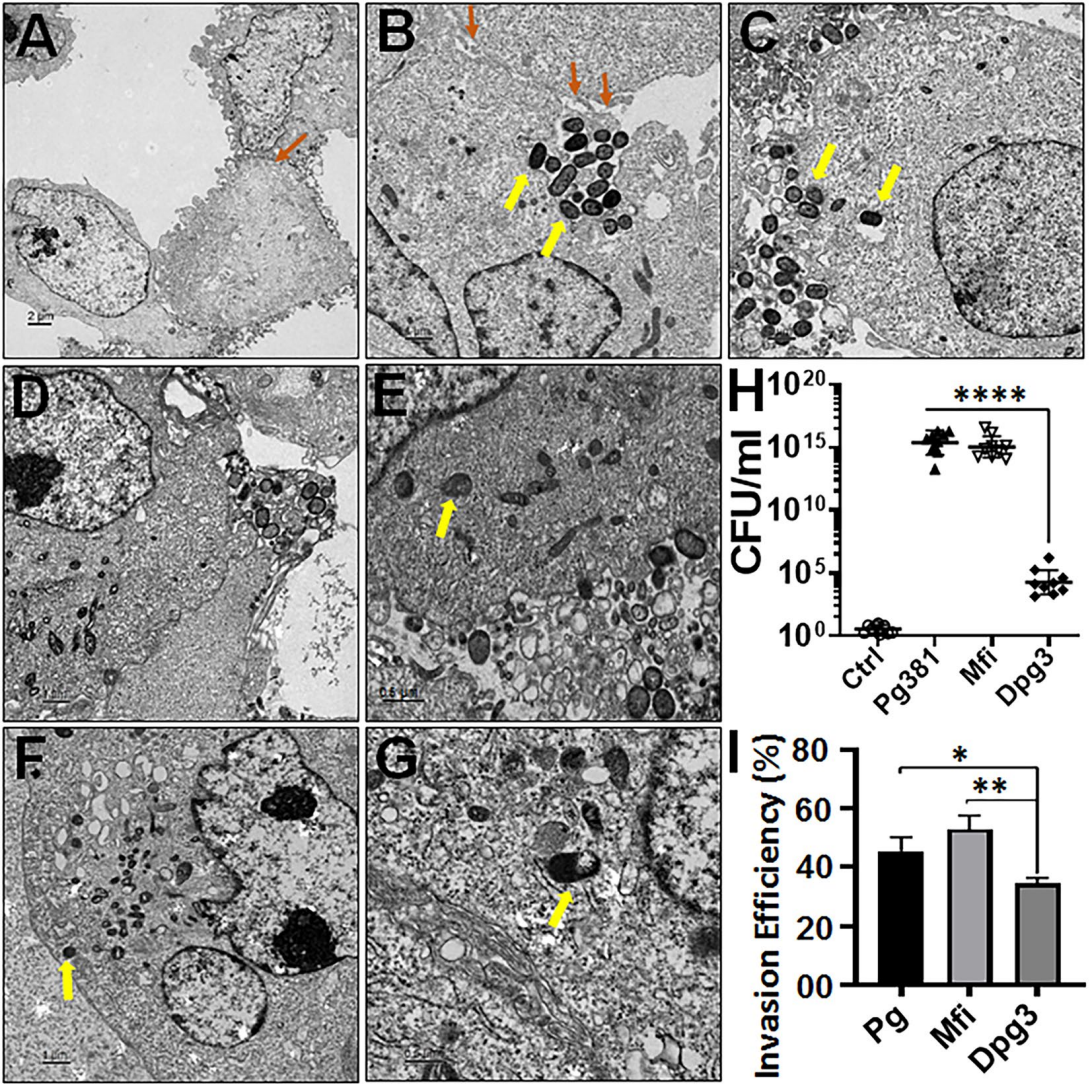
High internalization and intracellular content of *Pg* strains within ARPE cells. (**A–G**) Transmission electron microscopy (TEM) of human ARPE cells infected with all fimbriated *Pg strains* (10 MOI) for 24 hours. *P. gingivalis* and mutant strains internalized retinal pigment epithelial cells. The sections show the intra-and extra-cellular contents of un-infected control (**A**), *Pg*381 (**B,C**), MFI **(DE**) and DPG3 (**F,G**) infected ARPE cells for 24 hours with 10 MOI (2 sets of representative images from each group). Orange arrows indicate several epithelial junctions such as long and short tight junctions between ARPEs and loss of integrity (**B**). Yellow arrows indicate *Pg* strains freely occupy the cytoplasm (**B,C,E–G**). The top and bottom sections show the different magnifications of randomly selected sections (Scale bar:- A-B: 2 µm, C-E, G: 1 µm, F, H: 0.5 µm). Refer the low magnification in Fig. S5 for the complete view. (**H,I**) Antibiotic Protection Assay: The figure shows the survival of both adhered and internalized (**H**), and only internalized (**I**) *P. gingivalis* strains after 24 hours of infection. ARPE cells infected with *Pg*381 or isogenic mutant strains and incubated with or without antibiotics were lysed and the survived extra (cell surface/membrane attached) and intracellular bacteria were re-suspended and maintained in anaerobic broth for 3 days. (**H**) The data represents CFU within and on the surface of ARPEs harvested from biological triplicates before antibiotic treatment. The experiments were repeated thrice with three technical replicates (n = 9). The means ±standard deviation (in triplicates) were analyzed by One-way ANOVA of different groups and Tukey’s test for multiple group comparisons within three different experiments (n = 3) with 3 technical replicates (n = 3) from each experiments. (n = 9; ****P < 0.0001). CFU - Colony Forming Unit. (**I**) Invasion efficiency of *Pg* strains were expressed as the percentage of the initial inoculum recovered after antibiotic treatment. The means ± SD were analyzed by One-way ANOVA of different groups and Dunn’s test for multiple group comparisons with three different experiments with three technical replicates from each experiments. (n = 9; *P < 0.05; **P < 0.001). Refer Fig. S9 for adhesion and intracellular survival of *Pg* strains in AEPE cells.

Next, we tested whether the *Pg* mutant strains MFI and DPG3 were also able to invade ARPE-19 cells. The TEM results show the intimate interaction and internalization of *Pg* strains and presence of nano-sized cellular vesicles, presently undefined, in ARPE cells after exposure to *Pg*381, MFI and DPG3 at 10 MOI at 24-hours (Fig. 6B–G) compared with uninfected control (Fig. 6A). The uptake of all *Pg* strains by ARPE cells is more obvious and most evident with higher numbers of *Pg*381 (Fig. 6B,C and S5 A–D) and MFI (Fig. 6D,E and S5E,F) intra- and extracellularly compared with DPG3 (Fig. 6F,G and S5G,H). We also observed most of the *Pg* strains freely occupied the cytoplasm of ARPE cells (Fig. 6B,C,E–G) and released their intracellular content (Fig. S5C).

### **Adhesive and invasive abilities of***P. gingivalis***strains in the ARPEs**

The abilities of *Pg*381 and its isogenic mutant strains (MFI and DPG3) to adhere and/or invade ARPE cells was examined and further confirmed by an antibiotic protection-based recovery assay. Survival and persistence of viable adherent and/or intracellular bacteria (CFU) was then assessed quantitatively by lysing RPE cells and growing bacteria in broth cultures and on anaerobic blood agar plates (Fig. S9). *Pg*381 and MFI was significantly recovered at higher numbers from RPEs lysates in broth and on blood agar compared to DPG3 (Fig. 6H). This difference was most apparent after 24-hours, with large numbers of surface and intracellular bacteria present (Fig. S9). Consistent with their adherence capabilities, *Pg*381 and MFI, both of which express major *FimA* fimbriae, showed an increased invasive ability (Fig. 6H), suggesting a role for *FimA* in invasion of the RPE cells. Next, we estimated the invasion efficiency of viable bacteria recovered for each strain (Fig. 6I). Interestingly, measurement of the survival of internalized *Pg* (45%) and major fimbria mutant MFI (52%) showed significant invasive efficiency when comparing with the major fimbriae-lacking strain DPG3 (Fig. 6I), which showed approximately 30% survival within ARPE cells. These results imply an involvement of both *Pg*381, and MFI (*FimA*), in recognition of specific receptors on the surface of retinal pigmented epithelial cells. However, further investigation needed to elucidate the exact mechanisms.

### *P. gingivalis***microorganisms massively clustered along the ARPE cell surface adherent and cellular protrusions**

The TEM showed evidence of clustered *P. gingivalis* microorganisms along the ARPE cell surface, both at the apical and basolateral sides (Fig. S6A,B). Higher magnification showed adherent *Pg*381 (Fig. S6A1) and subsequent cellular protrusions (Fig. S6B1). We also observed the apparent massive contact between micro-filamentous cellular components and surface-adhering *P. gingivalis* (Figs. S6B1 and S7). As demonstrated in Fig. 6, numerous *P. gingivalis* organisms were evident extra and intracellularly. Most extracellular *P. gingivalis*, appear to be clustered at certain spots along the cellular membrane, while the rest of the membrane remained relatively free of bacteria (Fig. S6B). This could be due to *Pg* aggregation by fimbrial interaction with the presence of cellular receptors that are expressed only at certain areas on the surfaces of the cellular membrane and subsequent attachment and/or invasion by the aggregate as a whole.

### **Internalized***P. gingivalis***microorganism initiation or undergoing division and intracellular survival in ARPE-19 cells**

**Figure 7:**
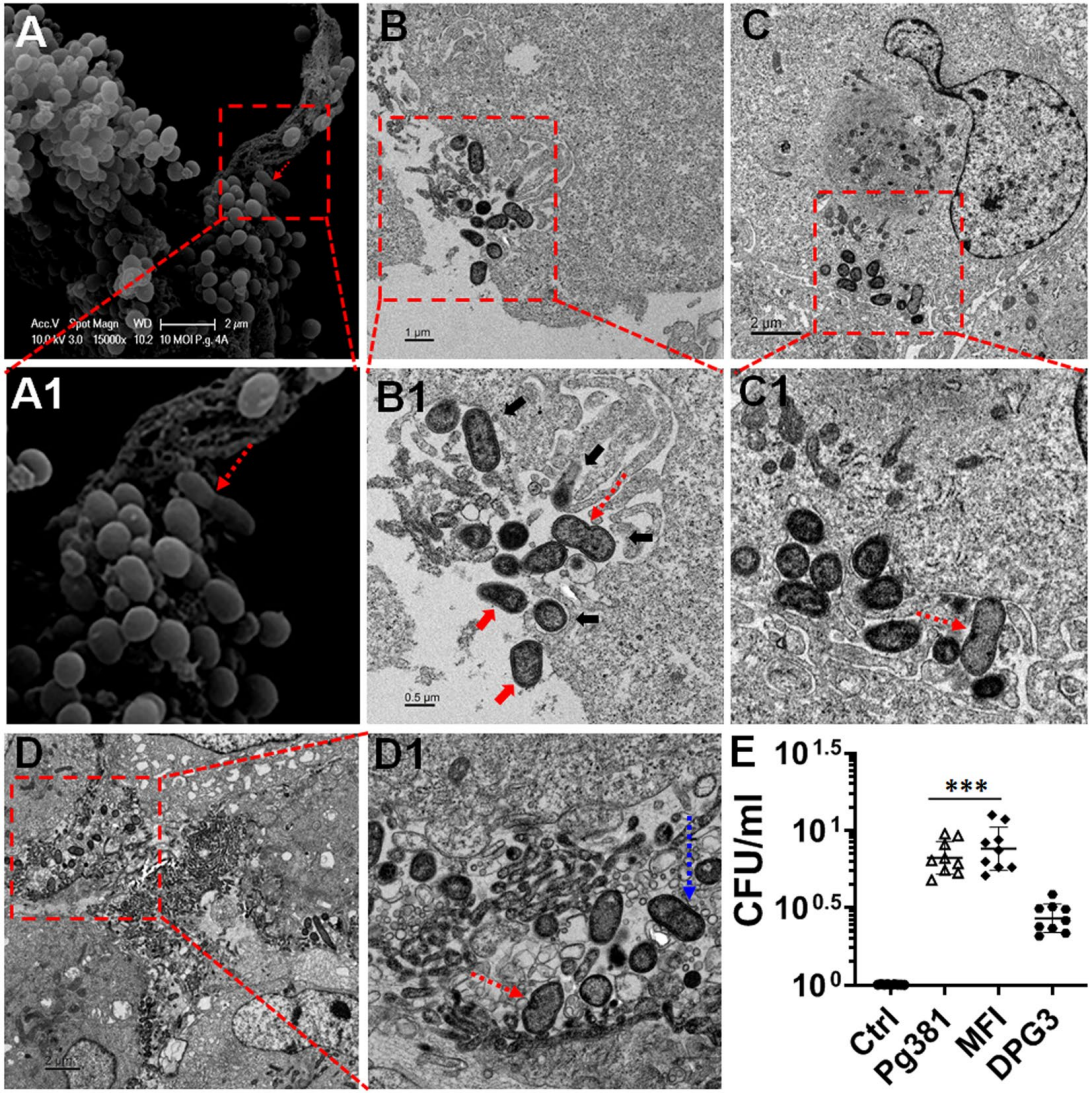
Internalized *Pg381 and MFI* microorganism initiation or undergoing division and intracellular survival in ARPE-19 cells. (**A**–A1) SEM showed evidence of *Pg* undergoing division on the surface of infected ARPEs at 10 MOI for 1 hour. (**B–D**) TEM images illustrate the internalized *Pg3*81 and MFI (10 MOI) microorganism initiation (dotted blue arrow) or undergoing (dotted red arrows) division and survival in ARPE for 1 (**B**, B1) and 24 (**C**-C1 **& D**-D1) hours is evident. We observed the apparent contact between micro-filamentous cellular components (black arrows) and surface-adhering *P. gingivalis* (red arrows) while undergoing division (**B**-B1) at 1 hour. Evidently, several of the TEM images show, *Pg*381 (**C**-C1) and MFI (**D**-D1) dividing within the ARPE cells at 24 hours. This indicates that the bacteria are metabolically active during invasion and may be able to persist in the ARPE cells for at least 24 hours. Note- Massive extracellular/intracellular bacteria, especially in the case of *Pg*381 and MFI, appear to be aggregated at certain spots along the cellular membrane and cytosol. Also refer the second set of low to high magnified TEM images in Fig. S7 for the complete view and confirmation. A1, B1, C1 and D1 shows a magnified/enlarged region of Boxed areas in **A**, **B**, **C** and **D**, respectively. (Scale bar:- A-D: 2 µm, B: 1 µm, B1: 0.5 µm and A1, C1, D1: enlarged from **A**, **C** and **D**, respectively). (**E**) The figure shows the replication of *Pg*-strains at 1-hour infection and 5 hours post invasion; the cells were lysed, *Pg381*, MFI and DPG3 recovered and CFU enumerated as described in the methods. The data represents CFU within ARPE cells harvested from biological triplicates. The mean ± SD were analyzed by One-way ANOVA of different groups and Tukey’s test for multiple group comparisons within 3 different experiments with 3 technical replicates from each experiments. (n = 9; ***P < 0.001). CFU- Colony Forming Unit. Refer Fig. S9 for *Pg* strains replication/colony formation.

SEM demonstrated adherent *Pg*381 (10 MOI) microorganisms undergoing apparent division in ARPE for 1 hour (Fig. 7A,A1), we thus further analyzed the TEM for *Pg-*fimbriated mutant’s multiplication at 1 and 24-hours. TEM confirmed that internalized *Pg*381 and MFI (10 MOI) microorganism initiation or undergoing division in ARPE for 1 (Fig. 7B-B1) and 24 (Fig. 7C,D) hours respectively, however, no cell division of DPG3 observed. There was apparent contact between micro-filamentous cellular components and surface-adhering *P. gingivalis* while undergoing division at 1 hour as visualized by SEM (Fig. 7A-A1) and TEM (Fig. 7B-B1).

We observed most (~60–70%) of the bacteria located at the apical membrane (Fig. 7A-A1) and the remaining near the basolateral margins (Fig. 7B-B1). Evident in several of the TEM images, are *Pg*381 (Fig. 7C-C1 and S7) and MFI (Fig. 7D-D1) dividing within the ARPE cells at 24-hours, which indicates that the bacteria were metabolically active during invasion and plausibly surviving inside the ARPE cells for at least 24-hours. Note that, at certain spots enormous extracellular/intracellular bacteria, especially *Pg*381 and MFI, appear to be aggregated along the cellular membrane and cytosol.

### **Intracellular replication of*Pg*strains within ARPE cells**

**Figure 8:**
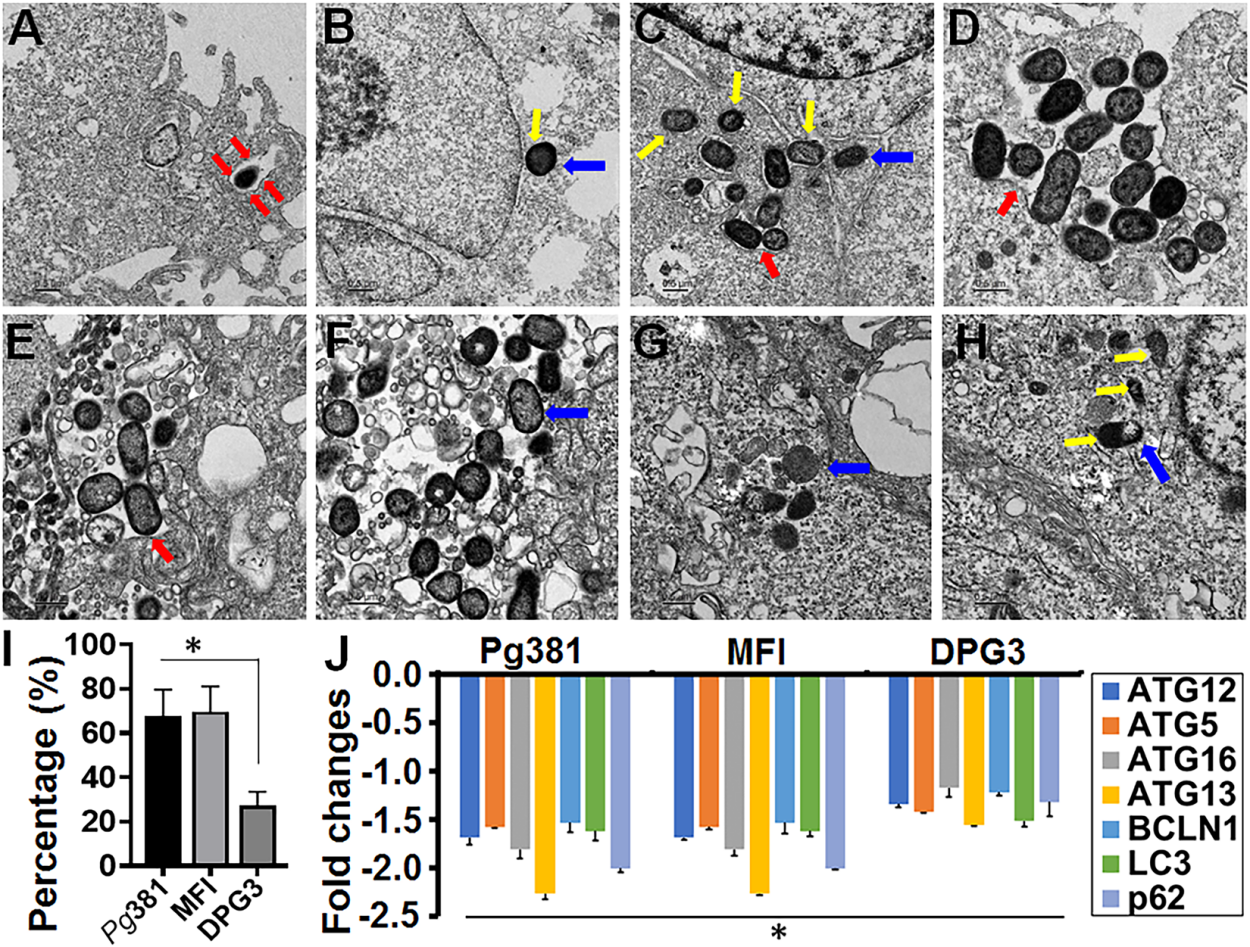
*P. gingivalis* enclosed within single membrane structures or freely occupy the cytoplasm of ARPEs and escaped the autophagic vesicles. (**A–H**) Transmission electron microscopy (TEM) of single membrane structures within ARPEs infected with *Pg*381 (1 & 10 MOI), MFI and DPG3 (10 MOI) for 1 and 24 hours. All *Pg* strains shows mostly escaped the autophagic (double membrane) vesicles and enclosed within single membrane structures (red arrows) or freely occupy the cytoplasm (blue arrows). Yellow arrows show the bacteria in the cytosol around the nucleus. (**A**) *Pg*381 invaded into ARPEs enclosed within single membrane structure is evident at 1 MOI for 1 hour, whereas it reached the nucleus and lived-in the cytoplasm at 24 hours (**B**). As hypothesized, *Pg*381 (**C,D**), and MFI (**E,F**) were consistently detected within single membrane vesicles or freely occupy the cytoplasm of retinal epithelial cells, in contrast to our hypothesis that the DPG3 (G, H) also enclosed within the characteristics single membrane structures and apparently observed in the cytoplasm freely and close to the nucleus (yellow arrows) at 10 MOI for 24 hours. We also observed smaller numbers of DPG3 than the *Pg*381 and MFI both intra and extra cellularly (2 sets of representative images from each group). (**I**) The ratio of intracellular bacteria included in the single membrane were compared to total number of bacteria within ARPEs and plotted as percentage. Counting of the bacteria included in single membrane vesicles, freely occupy the cytoplasm or vacuoles after 24 hours of infection. Each strain was counted in six randomly selected grids for each sample. The ratio of DPG3 trapped in single membrane relative to the total intracellular bacteria was significantly lower than *Pg*381 and MFI. Refer the low magnification in Fig. S8 for the complete view. *P < 0.02. The analysis of the bacterial counts used Kruskal-Wallis test of different groups and Dunn’s test for multiple comparisons with 3 different experiments. (Scale bar:- A-H: 0.5 µm). J) TaqMan quantitative PCR results shows the genes related to autophagy machinery are significantly decreased by *Pg*381 (*Mfa-1/FimA*), MFI (*FimA*) and DPG3 (*Mfa-1*) infected ARPE-19 cells for 24 hours compared with uninfected control. This data is consistent with TEM imaging analysis (Figs. 5–8A–I) and confirm the lysosomal/vacuolar escape mechanism of *P. gingivalis*. The experiments were repeated thrice (biological) with three technical replicates (n = 9). Fold change in gene expression was normalized to the control and ≥ ±1.5 fold was considered as significant (*P < 0.05).

In order to study the replication of the intracellular *Pg* and its mutants within RPE cells, we harvested intracellular *P. gingivalis* using the antibiotic protection assay at 1-hour infection and 5-hours post invasion. The result shows significant replication of *Pg* and MFI compared with DPG3 (Fig. 7E). We also observed a moderate increase in the MFI counts compared with *Pg*, but no significant difference. To our surprise, we detected intracellular DPG3 (*Mfa1*) amplification at 24-hours infection (Figs. 6I) and 5-hours post invasion (Fig. 7E), whilst we could not observe any dividing cells by TEM analysis as shown for *Pg* and MFI (Fig. 7). However, this data is consistent with all other imaging analysis (Fig. 6H,I and Fig. 8I), including confocal quantifications (Fig. 2F). Thus, *P. gingivalis* and its mutant strains appears to be capable of replication within the RPE cells, increasing in numbers significantly over a 5-hours period (Fig. 7E). This could be one of the potential strategy of escape mechanisms employed by *Pg* to defend from RPE cell immune responses.

### *P. gingivalis***escapes the autophagic vesicles and enclosed within single membrane structures or freely occupy the cytoplasm of retinal epithelial cells**

The presence of autophagic double membrane vesicles was examined by TEM analysis in ARPEs infected with *Pg* and fimbria mutant strains. Tracking of *Pg*381, MFI and DPG3 (10 MOI) within ARPEs after 24-hours of infections demonstrated that the majority of bacteria were contained in single membrane structures (Fig. 8A–H), with notable absence of autophagic vesicles. All *Pg* strains exhibited a cell wall structure with an outer and inner cell membrane typical of Gram-negative bacteria. The results illustrate that *Pg*381 invades into RPEs, enclosed within single membrane structures, evident at 1 MOI for 1-hour (Fig. 8A and S8A), while it reached the nucleus and was not degraded in the cytoplasm at 24-hours (Fig. 8B and S8B).We also noted, greater numbers of *Pg*381 (Fig. 8C,D), MFI (Fig. 8E,F) consistently detected within single membrane vesicles or freely occupying the cytoplasm of ARPEs compared with DPG3 (Fig. 8G,H). However, all the *Pg* strains demonstrated that they were freely occupying the cytoplasm (Fig. 6B–G). Quantification of the single membrane structures that contained bacteria in or freely occupied the cytoplasm were carried out in six randomly selected TEM images of each sample. The ratio of *Pg*381 and MFI trapped in single membrane relative to the total intracellular bacteria were significantly higher than DPG3 (Fig. 8I), which is consistent with confocal imaging (Fig. 2) analysis.

### **Autophagy escape mechanism of*Pg*-strains in ARPE-19 cells**

Our previous study has demonstrated the role of *Pg*-*Mfa1* and *FimA* in dendritic cells^35^, in killing or survival by autophagy evasion^39^. Here, TEM showed autophagy vesicle evasion of *Pg*-strains in ARPE cells (Fig. 5 and 8). To further corroborate the lysosome/vacuolar escape mechanism of *Pg*-strains through the deactivation of autophagy signaling pathway in RPEs cells, we assessed the expression of autophagy related (ATG) genes by qPCR. Expectedly, our results show that *Pg*-strains significantly (P < 0.01; ≥±1.5 fold) downregulated the family of ATG genes including those of the autophagosome and controlling complex ATG12-ATG5:ATG16L1, and LC3 (autophagy marker), ATG13, cargo selection molecule or ubiquitin-binding protein p62 (or Sequestosome-1/SQSTM1) and beclin-1, compared with uninfected control (Fig. 8J). This leads to deformed or immature autophagosome and reduced clearance of autophagic substrates in RPE by *Pg-*strains, which may cause RPE dysfunction and damage due to chronic exposure to this dysbiotic pathogen. Collectively, these data revealed that ARPEs infected with *Pg-*strains induced downregulation of autophagy related genes, supporting a mechanism for how *Pg* escapes host immune response and immune homeostasis disruption.

## Discussion

To our knowledge, this is the first report on the invasion of human retinal pigment epithelial cells by a dysbiotic oral-pathogen *P. gingivalis in-vitro* and its intracellular fate within the cytosol and single-membrane vacuolar compartments via autophagy evasion. Evidences indicate that *P. gingivalis* can invade several cell lines of diverse origin including gingival epithelial cells (GECs)^41^, endothelial cells^42^, dendritic cells^43^, macrophages^44^, osteoblasts^45^ and fibroblasts^46^. Studies suggest that there are positive relationships between periodontitis and tumors of Oro-digestive tract, pancreas and lung^47^. Our previous publication reported the virulence trait of *Pg* in potential oral epithelial-cell proliferation^35^ and other studies confirmed tumor-like transformation with long-term infection of *Pg*^48^. Active invasion of aortic tissues by *Pg* in mice causally links experimental periodontitis and atherosclerosis *in-vivo*, as *Pg* was found to be the most abundant*(80%)* species in atherosclerotic plaque^49^. Chronic systemic inflammation instigated by PD could be correlated with neuroinflammation as in Alzheimer’s disease, where *Pg* has been detected invading brain tissue through compromising the blood-brain-barrier^50–53^. In patients with both rheumatoid arthritis and PD, *Pg* DNA was detected by PCR in the synovial fluid^8,54^. This prompted us to surmise that periodontopathogens can instigate chronic inflammation at non-oral sites like eye by subversion of innate immune elimination through cellular invasion and activation of host-signaling pathways^35,55^. Hence, we intended to investigate the potential pathophysiological link between AMD and oral dysbiosis, using *P. gingivalis* as a prototype dysbiotic species^56^. This *in-vitro* study has demonstrated significant interaction of fimbriated-*Pg*381 with membrane of human retinal pigment epithelial (ARPE-19) cells (RPE in the following text), leading to invasion without obvious degradation of *Pg*381 within the cytosol and vacuolar structures.

*P. gingivalis* employ a variety of virulence factors, especially through the adhesive properties of its fimbriae, to invade the host cells and escape host immune surveillance^57,58^. The major *FimA* and minor *Mfa1* fimbriae of *Pg* play a pivotal role in adhesion to host cell surface. Immunofluorescence confocal imaging demonstrated significant uptake of *Pg*381 by RPE cells at different multiplicity of infection (MOI) which were apparently distributed in the cytosol and around the cell nuclei. Uptake of *Pg*381 occurred in a concentration dependent manner, with intracellular *P. gingivalis* observed into RPE cells even at 1 MOI. *P. gingivalis* adhered and tightly engaged the human retinal pigmented epithelial cell surface at 1-hour at both 1 and 10 MOI, while highly clustered and engaged at 10MOI. Significantly, live *Pg* invaded retinal epithelial cells but not heat-killed bacteria, indicating an active invasion as opposed to phagocytosis. We assume that, in the living animal, the apical side of the RPE is in contact with the subretinal space (minimal space just between the apical villi of the RPE and the ends of the interdigitating outer segments of the photoreceptor cells), which could be varying with the mode of potential interaction of RPE with *P. gingivalis* or their endotoxins released would likely come in contact with the basolateral/choroidal side of the RPE. Further, in a chronic inflammatory state, as is said to exist in AMD and would certainly arise congruent with prolonged exposure to the fimbriated dysbiotic pathogen *Pg*, the outer blood-retinal barrier may become compromised. Under this condition, the subretinal space (apical surface of RPE) could additionally become susceptible.

Certain bacteria exploit the intracellular environment as a type of ‘armor’ during initial stages of infection or until they develop strong virulence against the host tissue^40^. Consistent with these observations and as an evidence of early host-pathogen interaction or engagement, scanning electron microscope (SEM) showed *P. gingivalis* adhering and tightly engaging the human RPE cell surface at 1 hour (1 and 10 MOI) and highly clustered at 10MOI. Earlier studies have shown that *Pg* express vesicles on their cell surface which are released into its environment. *P. gingivalis* may employ these vesicles as a vehicle for imperative virulence factors and proteolytic enzymes, major components of outer membrane proteins which are responsible for destruction of periodontal tissues, initiating host immune response and host cell invasion^59^. This was apparent from our results as the intracellular *Pg* was found within vesicular structures with single enclosed membranes inside the retinal epithelial cell, without endocytic vacuoles surrounding them.

*P. gingivalis* is an unique periodontopathic bacterium capable of altering its local environment to its favor, modifying the host immune systems and facilitating other colonizers while setting up the platform for pathogenesis^20^. Evidently, our SEM and TEM (transmission electron microscopy) data together confirmed that not only *Pg*381 but also the mutant strains were able to enter RPE cells. *P. gingivalis* showed signs of division as there was apparent contact between surface adhering *Pg* and micro-filamentous cellular components. Previous studies have shown that *Pg* in GECs, make use of actin-dependent protrusions to spread to the new host cells and propagate efficiently evading the host defense^60^. *P. gingivalis* promotes its adaptive fitness by evading clearance *in-vitro* and *in-vivo*. The unique ability of pathogens to evade lysosomal degradation is crucial to survive inside the host cells and induce persistent infections. A direct evidence of the intracellular survival of *Pg* has also been demonstrated in normal and immortalized human GECs^19^. Consistent with these previous findings, TEM revealed that *Pg* and its isogenic mutants (MFI and DPG3) invaded human RPE cells. The intracellular entry and survival of *P. gingivalis* and its isogenic mutants was quantitated by antibiotic protection/survival assays, which further confirmed the invasion efficiency and amplification of these strains. We found that viable *Pg* strains were recoverable in large numbers post-infection over a 5 and 24-hours period. We predict that this could be an escape mechanism employed by *Pg* to evade the cell immune responses. Thus, our data suggest that *Pg* not only survives in the cytosol of epithelial cells but also undergoes division. Furthermore, it has been previously shown that *Pg* is able to survive within DCs by subversion of canonical pathway via its *Mfa-1* fimbriae^39^. However, presently unclear is how the minor fimbriated strain DPG3 invades the retinal epithelial cells, and if the entry is fimbria-receptor mediated or through engulfment by cellular protrusion; further study is warranted in this regard.

Our previous studies^35,39^ and the current TEM data, lead us to test our hypothesize that the lysosome/vacuolar escape or survival mechanism of *Pg*-strains might be through the deactivation of autophagy signaling molecules in RPEs cells. Expectedly, qPCR results are consistent, showing significantly reduced expression of autophagy related genes upon infecting RPEs with *Pg-*strains, suggesting inhibition of the autophagy pathway. Autophagy has essential role in periodontal cell biology, bacterial internalization and elimination and immune-suppression, thereby inhibiting apoptosis in the periodontal cells, thus having a major role in the pathogenesis of PD^61,62^. Studies have determined the critical function of autophagy-lysosome system in the maintenance of normal homeostasis in the aging or senescent RPE^63^. Any disparity in this regulated cell clearance process is associated with age-dependent degeneration of RPE cells along with secondary degeneration of photoreceptors, critical for visual function^64^. Experimental AMD models have illustrated the formation of retinal atrophic patches, subretinal migration of microglial cells, sub-RPE drusen and drusenoid deposition, complement activation and sites of choroidal neovascularization^65^, typical of AMD. It is observed that autophagic potential declines with aging and is often found to be associated with down-regulation of autophagy-induction proteins namely ATGs, Beclin-1 and LC3 (most widely used marker of autophagosomes)^63^. Interestingly, Atg13 required for phagophore formation, was significantly downregulated in *Pg* (*Mfa-1/FimA*), MFI (*FimA*) and DPG3 (*Mfa-1*) infected RPEs. The conjugated Atg5–Atg12 complex that pairs with Atg16L dimers to form a multimeric Atg5–Atg12–Atg16L complex and associates with the extending phagophore, were also significantly downregulated by *Pg*-strains. In addition, beclin-1, an autophagic protein, important for nucleation of autophagy and regulation of apoptosis process^66^ was downregulated. The sequestosome-1 (p62/SQSTM1) is a significant factor in inflammatory response, autophagy and cellular senescence and p62/SQSTM1 can be a target for inhibition of retinal inflammation, improvement of autophagy and the antiaging of RPE cells^64^. p62/SQSTM1 binds autophagy substrates to the autophagosome by interacting with ubiquitinated proteins via its ubiquitin-associated domain^67^ and LC3 with its LC3‐interacting region (LIR)^68^. While p62 accumulation and association with protein aggregates is related to autophagy defect, p62 mutations is directly linked to selective autophagy impairment and in turn to neurodegeneration. Notably, our data demonstrated that *Pg*-strains significantly downregulated both p62 and LC3 genes in RPEs. These data collectively suggests that *Pg* applies lysosomal/vacuolar escape mechanism through dysfunctional autophagy for its persistent survival inside the RPE and may further mediate pathological effects i.e. immune homeostasis disruption in the retina upon chronic exposure, yet more investigation needed.

In conclusion, these findings altogether indicate that the dysbiotic periodontal pathogen *P. gingivalis* efficiently invade retinal epithelial cells in high levels, replicate and are sustained within them. This invasion and autophagy evasion by the keystone species may be one of the contributing elements in the pathogenesis of retinal degenerative diseases. However, it remains to be verified whether *P. gingivalis* invade RPE cells *in-vivo* in patients with AMD and PD, and via what route.

## Materials and Methods

### Reagents

Human Retinal Pigment Epithelial (ARPE-19) cell lines were obtained from ATCC (Manassas, VA). DMEM/F12 culture medium, fetal bovine serum, penicillin and streptomycin were from Life Technologies (Grand Island, NY). Gentamicin, Metronidazole, 5(6)-Carboxyfluorescein diacetate succinimidyl ester (CFSE; Molecular Probes), Rhodamine-phalloidin (F-actin), Trypsin (0.25%)/EDTA (0.1%), TRIzol reagent, High-Capacity cDNA Reverse Transcription Kit, TaqMan gene expression assay (primers/probes) and 8-well chamber slides were obtained from Thermo fisher scientific (Waltham, MA). RNeasy kit was from Qiagen (Germantown, MD). The universal power block was obtained from Biogenex Laboratories (Fremont, CA). DAPI (4′,6-Diamidino-2′-phenylindole dihydrochloride) from Sigma-Aldrich Co. LLC (St. Louis, MO).

### ***Porphyromonas gingivalis*and mutant strains culture**

*Porphyromonas gingivalis* 381 (wild-type *Pg*381), which expresses both minor (*Mfa1*) and major (*FimA*) fimbriae, isogenic major fimbria-deficient mutant *Pg*-DPG3 (DPG3), which expresses only the minor fimbriae (*Mfa1*^+^/*FimA*^−^) and the isogenic minor fimbria-deficient mutant *Pg*-MFI (MFI), which expresses only the major fimbriae (*Mfa1*^−^/*FimA*^+^) were maintained anaerobically (10% H_2_, 10% CO_2_ and 80% N_2_) in a Coy Laboratory vinyl anaerobic system glove box at 37 °C in Difco anaerobe broth MIC as we described earlier^35,69^. Erythromycin (5 µg/ml) and tetracycline (2 µg/ml) were added as per the selection requirements of the strains^35,39,70^.

### Human Retinal Pigment Epithelial (ARPE-19) cell culture

Human ARPE-19 has structural and functional properties distinctive of RPE cells *in-vivo* and this cell line will be valuable for *in-vitro* studies of retinal pigment epithelium physiology^71^. ARPE-19 cell lines were cultured and maintained in DMEM/F12K complete growth medium supplemented with 10% fetal bovine serum, 100 units mL^−1^ penicillin, and 100 μg mL^−1^ streptomycin and grown for 4–6 weeks and the cells were used after fifth passage for experiments as we described previously^72^. Briefly, the cells were grown to ~70–80% confluence then in serum-starved media per experimental conditions. After serum-starvation, fresh culture medium was then added to cells with and without bacterial strains at different multiplicity of infection (MOI) and different time points based on the experimental conditions.

### *P. gingivalis***strains labeling and Human ARPE-19 Infection**

Bacterial suspensions were washed thrice and re-suspended in PBS for spectrophotometer reading of 0.11 for optical density at 660 nm (OD_660_), which was previously determined to be equal to 5 × 10^7^ colony-forming units^69,73^. Corresponding bacterial counts were calculated and dilutions were prepared to infect ARPEs at different multiplicity of infection (MOI). To inactivate (heat-killed) bacteria, *P. gingivalis* was incubated for 1 hour at 95 °C prior to the experiment. For bacterial CFSE staining, the suspension was washed thrice and re-suspended in 5 μM of CFSE in PBS before being used as described earlier^39^. The bacteria were incubated for 30 min at 37 °C in the dark. Then the labelled *Pg* strains were washed twice with PBS and the residual CFSE dyes removed. Confluent (~70%) monolayers of ARPE-19 cells were then infected with *Pg* strains and placed into the anaerobic chamber at 37 °C and the pre-labeled *Pg* strains allowed to interact with host cells for 30–60 min. For infection assay, ARPEs were co-cultured with pre-labeled *Pg*381, *Mfa1*^+^*Pg*, and *FimA*^+^*Pg* at 1, 10 and 100 MOI in DMEM/F12K medium without antibiotics at 37 °C in a water-saturated atmosphere and incubated with the ARPEs for 1 or 24 hours and each experimental condition were performed in triplicate and repeated thrice.

### Immunofluorescence and Confocal Imaging Analysis

Human ARPE-19 cells were plated at a density of 1 ×10^4^ cells/well onto 8-well chamber slides 1 day before infection. At 70% confluence, the cells were infected with *Pg* and its isogenic mutant strains. 1). To test the uptake of bacteria by ARPE cells were infected with *Pg* at 1, 10, and 100 MOI (place chamber slides with infected ARPEs into anaerobic incubator and allowed bacteria to interact with host cells for 30/60 min) and incubated for 24 hours. 2). To determine the presence, internalization, colocalization and persistence of intracellular bacteria, the ARPE cells were infected with live or heat-killed CFSE labeled *Pg*, MFI and DPG3 for 24 hours and then stained with rhodamine-phalloidin (F-actin) for cytoskeleton visualization and DAPI, as described^74^. Briefly, after fixing and permeabilizing, the cells were blocked with 1X power block and incubated for 30 min at room temperature. To examine the colocalization of CFSE-labeled bacteria, ARPE-19 cells were stained with F-actin for 60 min before examination. After washing with PBS twice, subsequently, the slides were mounted after staining with the nuclear probe DAPI and the cells were examined by live-imaging by laser-scanning confocal microscopy (Carl Zeiss, Germany). Images were taken at different focal points (z-sections) for temporal-spatial visualization of bacteria.

### Adhesion and invasion antibiotic protection assay

The adhesive and invasive ability of *P. gingivalis* and its isogenic mutants in ARPE-19 cells were determined by the standard antibiotic protection assay as described^41,75^,with some modification. Briefly, cells were seeded in 6-well flat-bottom culture plates at a cell density of 1 × 10^6^ cells per well and grown overnight. Cells were infected with *Pg*381, MFI and DPG3 at 10 MOI and incubated for 24 hours. For determining total adhesion and invasion levels of *Pg* strains, the cells were washed thrice in PBS, re-suspended in sterile water on ice for 20 min to lyse the cells, with mechanical scraping and agitation to release the internalized bacteria. For invasion assay, external, nonadherent bacteria were removed by washing three times in anaerobic PBS, and external adherent bacteria were then killed by incubating for 1 hour with DMEM:F12 media containing 300 µg/ml of gentamicin and 200 µg/ml of metronidazole, and the cells lysed. Cell lysates were re-suspended in anaerobic broth for three days. After broth incubation, bacterial suspensions were washed thrice in PBS and re-suspended for spectrophotometer reading at OD_660_ in triplicate. The experiments were repeated thrice with three technical replicates. Viable counts (CFU) were enumerated based on a plate count serial dilution versus OD readings. For confirming the identity of the *P. gingivalis* (black pigmented Gram-negative coccobacilli), suspensions were cultured on 5% blood agar plates in triplicate under anaerobic conditions (10% H_2_, 5% CO_2_ and 85% nitrogen). Plates were then incubated in anaerobic conditions at 35 °C for 5 days until black colonies were detected.

### **Replication and survival assay of***P. gingivalis***strains in human ARPE-19 cells**

Additional experiments were conducted to address the possibility of intracellular replication and survival as described^41,76^ with minor modification. Briefly, *P. gingivalis* strains were incubated with ARPE cells for 1 hour and the extracellular bacteria were killed with antibiotics (300 µg/ml of gentamicin and 200 µg/ml of metronidazole). After washing to remove the antibiotics, the cells were maintained for a further 5 hours to allow replication of intracellular bacteria. Antibiotics were added again to kill any released extracellular bacteria and cells were washed thrice in PBS and re-suspended in sterile water on ice for 20 min to lyse the cells. Cell lysates were re-suspended in anaerobic broth for three days. Bacterial suspensions were washed three times in PBS and re-suspended for spectrophotometer reading at OD_660_ in triplicate. The experiments were repeated thrice with three technical replicates. Corresponding CFU counts were calculated based on a linear regression of plate count in serial dilution versus OD readings. Black colonies were confirmed in blood agar plate under anaerobic conditions (10% H_2_, 5% CO_2_ in nitrogen).

### Transmission Electron Microscopy (TEM)

ARPE-19 cells were plated into 6 wells 1 day before infection and when at ~70% confluence cells were infected with *P. gingivalis* or its isogenic mutant strains at 1 or 10 MOI for 1 and 3 hrs. The infected cells were then harvested and fixed or after further culturing in fresh medium without antibiotics for 24 hours. After ARPEs fixation, the procedures were carried out as described ealier^39^. Briefly, following incubation with bacteria, monolayers were washed three times in PBS and detached by trypsinization. The cell pellets were washed with PBS thrice and fixed in 2% glutaraldehyde in 0.1 M sodium cacodylate (NaCac) buffer, pH 7.4, post-fixed in 2% osmium tetroxide in 0.1 M NaCac, stained en bloc with 2% uranyl acetate, dehydrated with a graded ethanol series and embedded in Epon-Araldite resin. Thin sections were cut with a diamond knife and stained with uranyl acetate and lead citrate. Cells were observed in transmission electron microscope (JEM 1230—JEOL USA Inc.) at 110 kV and imaged with a CCD camera and first light digital camera controller (Gatan Inc.). ARPE cells containing *Pg* strains were photographed at each time point.

### Scanning Electron Microscopy (SEM)

ARPE-19 (1 × 10^5^) cells were grown on cover-glass in 24-well plates, incubated with *Pg* at 1 and 10 MOI for 1 hour, and bacteria attached to retinal epithelial cells were observed with SEM as we previously described^39^. Briefly, the cells were fixed for 30 min in 4% paraformaldehyde, 2% glutaraldehyde in 0.1 M sodium cacodylate (NaCac) buffer, pH 7.4, post-fixed in 2% osmium tetroxide in NaCac buffer, dehydrated with a graded ethanol series (25–100% for 5 min each), followed by a graded alcohol hexamethyldisilazane (HMDS), and HMDS was allowed to evaporate overnight in a fume hood. The dried discs were mounted on aluminum stubs with carbon adhesive tabs and sputter coated with gold-palladium. Discs were observed and imaged in a FEI XL30 scanning electron microscope (FEI, Hillsboro, OR) at 10 kV.

### RNA isolation and TaqMan-qPCR Assay

Total RNA was isolated as we described previously^35,69^ using TRIzol reagent and purified with RNeasy kit from uninfected control, and *Pg*, DPG3 & MFI (at 10MOI) infected ARPEs were cultured in 6-wells plates for 24 hours. RNA quantity and integrity were tested and only ratios of absorbance at 260 and 280 nm of 1.8–2.0, were included in this study. Analysis of gene expression in ARPEs induced by *Pg*-strains was performed using RT-PCR as we described previously^35^. Complementary DNA was synthesized from 1.0 μg RNA through a reverse-transcription reaction (Applied Biosystems). Real-time quantitative PCR was performed on TaqMan gene expression primers fast plates in triplicates for selected genes: ATG5 (Assay ID: HS00169468_m1), ATG12 (Assay ID: HS1047860_g1), ATG13 (Assay ID: HS00521135_m1), ATG16L1 (Assay ID: HS01003142_m1), LC3 (Assay ID: HS01061917_g1), Beclin-1 (Assay ID: HS1007018_m1), p62/SQSTM1 (Assay ID: HS01061917_g1) and GAPDH (Assay ID: HS02758991_g1). Three experimental (technical) replicates were analyzed for each biological sample and repeated thrice. The calculations for fold regulation used the 2^−ΔΔCt^ method as described in our previous report^69^ and GAPDH were used as internal controls.

## Supplementary information

Supplementary information.

## Data Availability

The datasets generated and analyzed during the current study are available from the corresponding authors on reasonable request.
