# Supplementary material for: Invasion of Human Retinal Pigment Epithelial Cells by Porphyromonas gingivalis leading to Vacuolar/Cytosolic localization and Autophagy dysfunction In-Vitro
Source: Sci Rep. 2020 May 4;10:7468. doi: 10.1038/s41598-020-64449-8 (PMC7198524; doi:10.1038/s41598-020-64449-8)

Invasion of Human Retinal Pigment Epithelial Cells by *Porphyromonas gingivalis* leading to Vacuolar/Cytosolic localization and Autophagy dysfunction *In-Vitro*

Pachiappan Arjunan[1*], Radhika Swaminathan[1], Jessie Yuan[1], Mohamed Al-Shabrawey[2], Diego G. Espinosa-Heidmann[3], Julian Nussbaum[3], Pamela M. Martin[4] and Christopher W. Cutler[1]

[1]Department of Periodontics, Dental College of Georgia, [2]Department of Oral Biology and Diagnostic Sciences, [3]Department of Ophthalmology, [4]Department of Biochemistry and Molecular Biology, Augusta University, Augusta, GA 30912, USA.

* **Corresponding author**:

Pachiappan Arjunan PhD.

Department of Periodontics

Dental College of Georgia, Augusta University,

Augusta, Georgia, USA.

Phone #: 706-446-5176; E-mail: PARJUNAN@augusta.edu

Supplementary Information

SI Figure S1. Heat killed *P. gingivalis* and its isogenic mutant strains do not invade Human Retinal Pigment Epithelial (ARPE-19) cells. (A-C) ARPE-19 cells were co-cultured with live CFSE-labeled *Pg*381, MFI and DPG3 (10 MOI) or heat killed (HK) all fimbriated *Pg* strains for 24 hours and compared to uninfected control. After fixation and permeabilization, the infected ARPE cells were stained with rhodamine-phalloidin (F-actin for cell surface) and DAPI (nuclear stain) and then examined by confocal microscopy. Representative images show the heat-killed *Pg381* (A), HK-MFI (B) and HK-DPG3 (C). Red - F-actin; Green - CFSE; Blue - DAPI. The data shown are representative of three similar results. Scale bar: 20 µm. Note:- These are the alternative and replicated images representing independent channels with respect to Figure 1.

SI Figure S2. Interaction of *P. gingivalis* with ARPE cell membrane. (**A-D**) The representative scanning electron microscopy (SEM) images show the primary interaction of *Pg*381 with the infected human ARPE-19 cells, for 1 hour with 1 and 10 MOI. Replicative mages of SEM at 1 (A, B) and 10 (C, D) MOI of *Pg*381 for 1hour infection. Note:- *P. gingivalis* were highly clustered and engaged with ARPE-19 cell membrane at 10 MOI (C and D). Scale bar: A-D: 10µm.

SI Figure S3. Highly clustered interaction and invasion of *P. gingivalis* within ARPE cells. (A-C) Transmission electron microscopy (TEM) of ARPE-19 cells infected with *P. gingivalis* at 1 (B and B1) and 10 (C and C1) MOI for 1 hour compared with uninfected control (A). These images show the clear host pathogen interaction and invasion of *Pg*381 (B – C) in ARPE cells relative to uninfected (A) control. Boxed areas in B and C show an enlarged/magnified region B1

and C1, respectively. (B1) Enlarged section showing *P. gingivalis* adhering to ARPE cell surface clearly. We observed signs of internalization established through fimbrial-adhesin–receptor complexes (blue arrows) with ARPE cells and formation of phagocytic cup like structures as well. (C-C1) *P. gingivalis* shows highly clustered internalization and freely occupied the cytoplasm of ARPE cells. This is consistent with the similar observation of SEM (S. Fig. C, D). Orange arrows indicate several epithelial junctions such long and short tight junctions between ARPEs. Scale bars:- A-C: 2µm, B1, C1: 1µm). Results are representative of four experiments (n=4). Note:- these are another or replicative set of representative images for figure 4.

SI Figure S4. *P. gingivalis* manipulation of ARPE cell invasion and escape to cytosol. (A-D) Representative TEM images of human ARPE cells infected with *P. gingivalis* at 1 MOI for 24 hours compared with uninfected control. (A) Another confirmative image for uninfected control (orange arrow shows the tight junction). (B-D) Representative TEM images for the low magnification of figure 5 (boxed areas in B, C and D magnified as shown in C, G and H, respectively in Figure 5) for the complete view. (B) Invaded *Pg* freely occupy the cytoplasm of ARPE cells (refer figure 5C for high magnification for boxed area of this image). (C, D) The adhesive properties of fimbriae allow *P. gingivalis* to invade ARPE cells and evade the host immune surveillance (refer figure 5G and H, respectively, for high magnification for boxed area of these images). (Scale bar:- A, B and D: 2µm, C: 1µm). Refer figure 5 for high magnification.

SI Figure S5. Internalization and intracellular content of *Pg* strains within ARPE cells. (A-H) Representative TEM images of human ARPE cells infected with Pg and its isogenic mutant strains at 10 MOI for 24 hours. The sections show the intra-and extra-cellular contents of *Pg*381

(A-D), MFI (E, F) and DPG3 (G, H) infected ARPE cells for 24 hours with 10 MOI (two sets of representative images from each group). (C) The internalized *Pg* release their intracellular content (IC), which can be seen clearly. Yellow arrows indicate *Pg* strains freely occupy the cytoplasm (D-H). Orange arrows indicate several tight junctions between ARPE cells. Boxed areas in D-H show magnified region as D-H, respectively in figure 6. The top and bottom panels show the different magnifications of randomly selected sections (Scale bar:- A-B: 1µm; C: 0.2µm; D-H: 2µm). Refer figure 6 for high magnification.

SI Figure S6. *P. gingivalis* microorganism massive cluster along the ARPE cell surface and cellular protrusions. (A-B) TEM shows the clustered *P. gingivalis* microorganisms along the ARPE cell surface. Higher magnification showing adherent *Pg*381 (A1, blue arrows) and subsequent cellular protrusions (B1, purple arrows). Boxed areas in A and B show a magnified/enlarged region as A1 and B1, respectively. (B1) Note the apparent massive contact between micro-filamentous cellular components (black arrows) and surface-adhering *P. gingivalis* (red arrows). (Scale bar:- A: 2µm, A1: enlarged from A1, B: 1µm, B1: 0.5µm).

SI Figure S7. Internalized *Pg*381 microorganism initiation or undergoing division. (A, B) Representative TEM images shows the second set of low to high magnified sections for the complete view and confirmation for figure 8. TEM images demonstrate the internalized *Pg*381 (10 MOI) microorganism initiation or undergoing division (blue arrow) and survival in ARPE for 24 hours is obvious. Markedly the TEM images, *Pg*381 show dividing within the ARPE cells at 24 hours (A2). Note:- Enormous intracellular *Pg*381, appear to be aggregated in the cytosol. (B) Internalized *Pg*381 shows the initiation of division as well as freely occupied in the

cytoplasm (B2 - enlarged view) of ARPEs are evident. (Scale bar:- A and B: 2µm, Boxed area in A and A1: enlarged to A1 and A2, respectively; B and B1: enlarged to B1 and B2, respectively).

SI Figure S8. *P. gingivalis* and its mutant strains enclosed within single membrane structures or freely occupy the cytoplasm of ARPEs and escaped the autophagic vesicles. (A-I) TEM of single membrane structures within ARPEs infected with *Pg*381 [1 (A-C) & 10 (D-E) MOI], MFI (F, G) and DPG3 (H, I) (10 MOI) for 1 and 24 hours. Red boxed areas in A, B, D, E, F, G, H and I show magnified regions as A, B, C, D, E, F, G and H, respectively, in figure 8. *Pg*381 and its isogenic mutant strains shows mostly exiting the autophagic (double membrane) vesicles and enclosed within single membrane structures or freely occupy the cytoplasm (blue arrows). Refer the figure 8 for corresponding magnified images and details. (Scale bar:- A, B, D and F: 1µm; C: 0.5µm; E, G, H and I: 2µm).

SI Figure S9. Schematic representation for adhesion and intracellular survival of *Pg* strains in ARPE-19 cells.

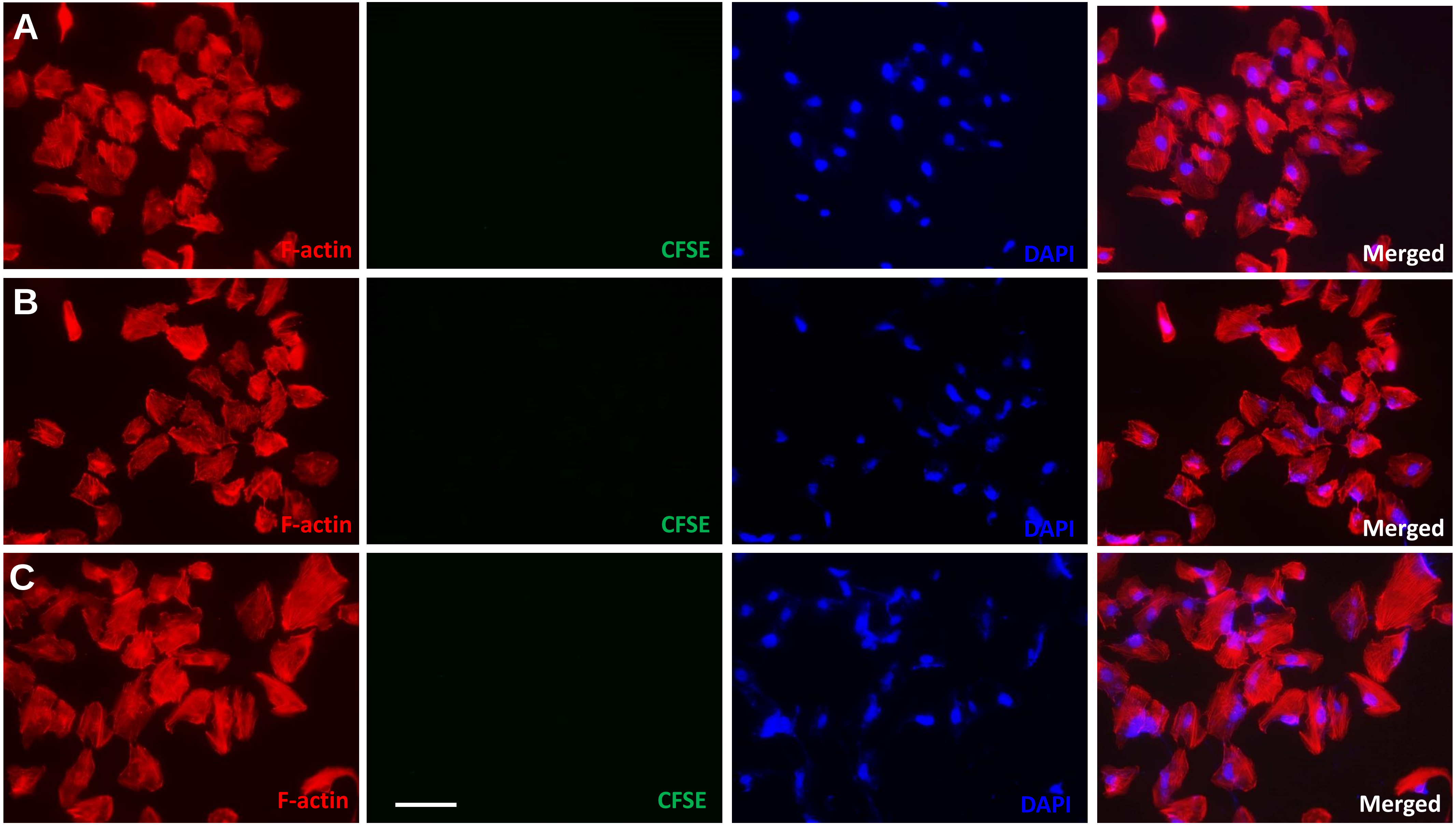
A
F-actin
CFSE
DAPI
Merged
B
F-actin
CFSE
DAPI
Merged
C
F-actin
CFSE
DAPI
Merged

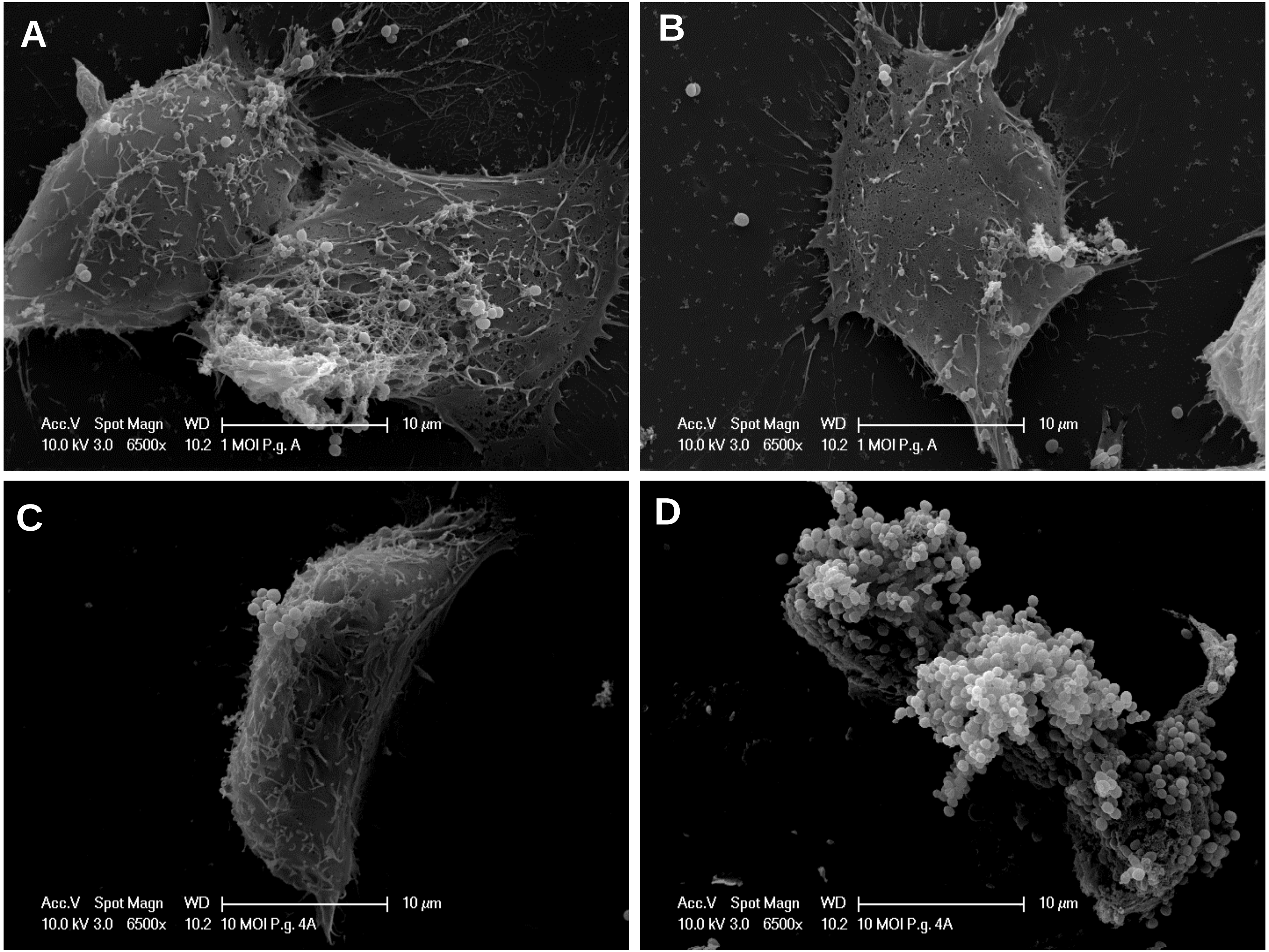
A
Acc.V Spot Magn WD 10 μm
10.0 kV 3.0 6500x 10.2 1 MOI P.g. A
B
Acc.V Spot Magn WD 10 μm
10.0 kV 3.0 6500x 10.2 1 MOI P.g. A
C
Acc.V Spot Magn WD 10 μm
10.0 kV 3.0 6500x 10.2 10 MOI P.g. 4A
D
Acc.V Spot Magn WD 10 μm
10.0 kV 3.0 6500x 10.2 10 MOI P.g. 4A

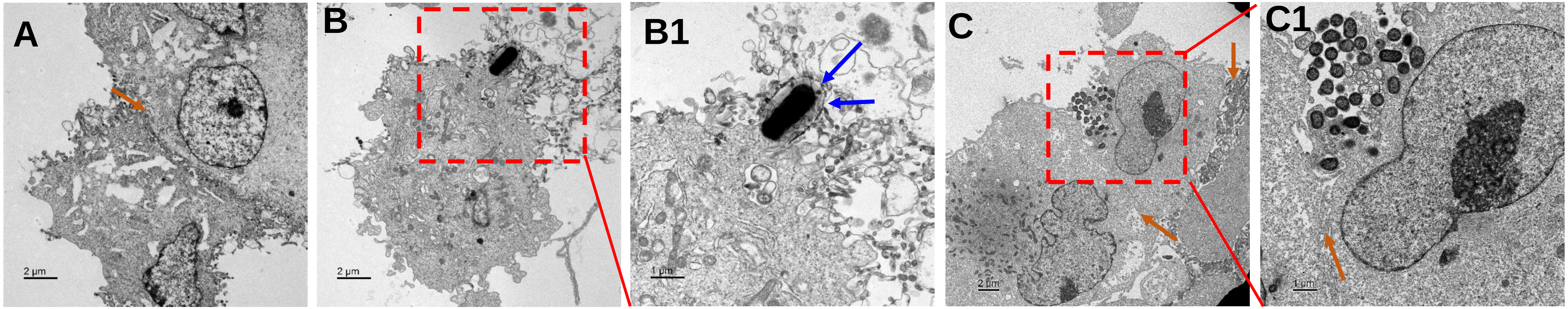
A
2 μm
B
2 μm
B1
1 μm
C
2 μm
C1
1 μm

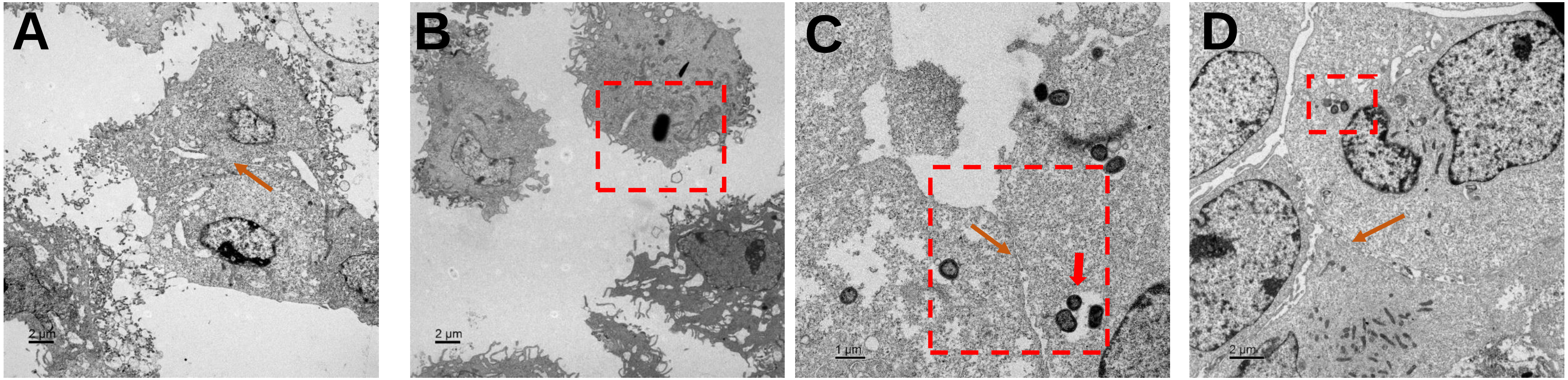
A
2 μm
B
2 μm
C
1 μm
D
2 μm

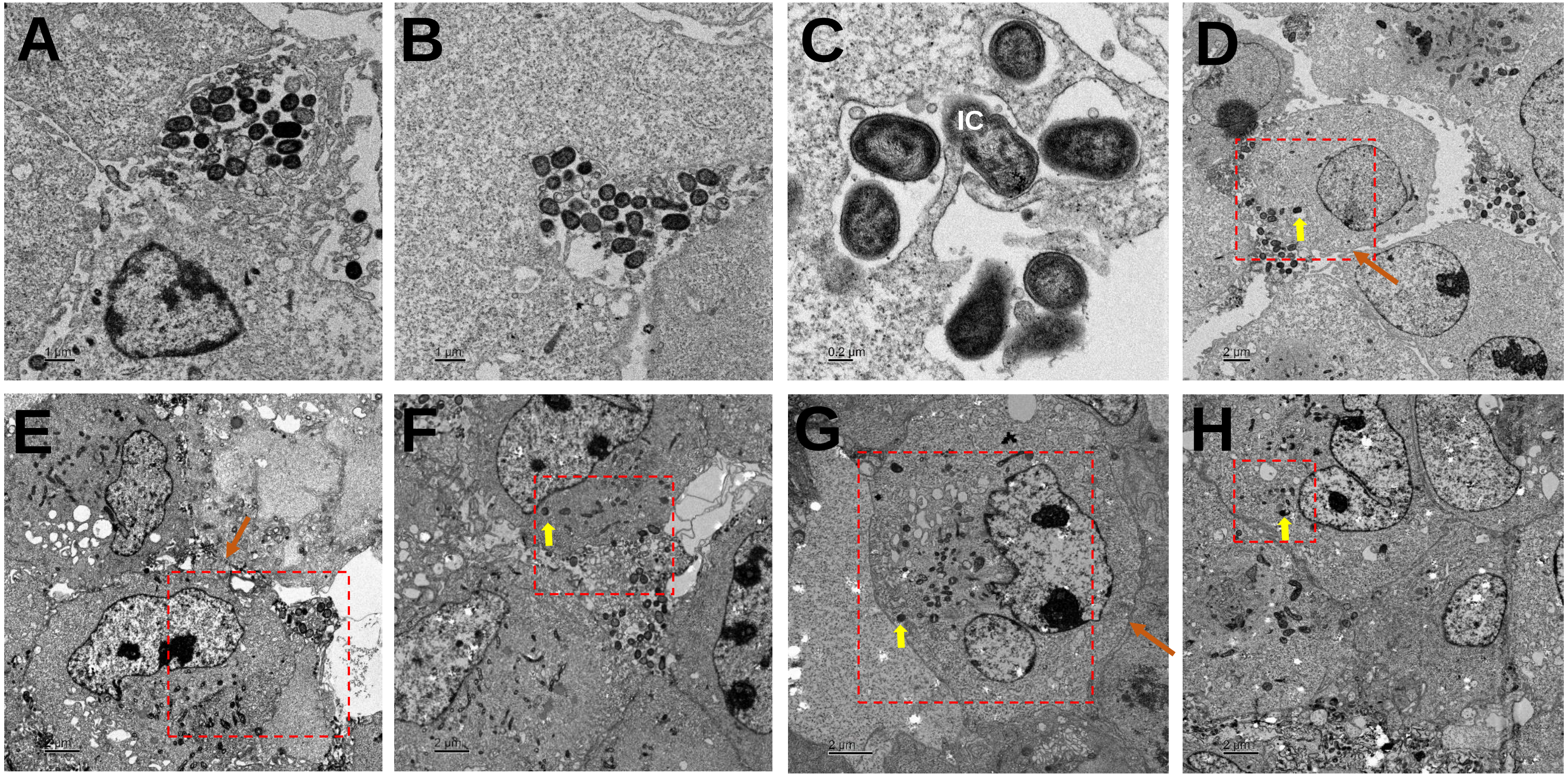
A
1 μm
B
1 μm
C
IC
0.2 μm
D
2 μm
E
2 μm
F
2 μm
G
2 μm
H
2 μm

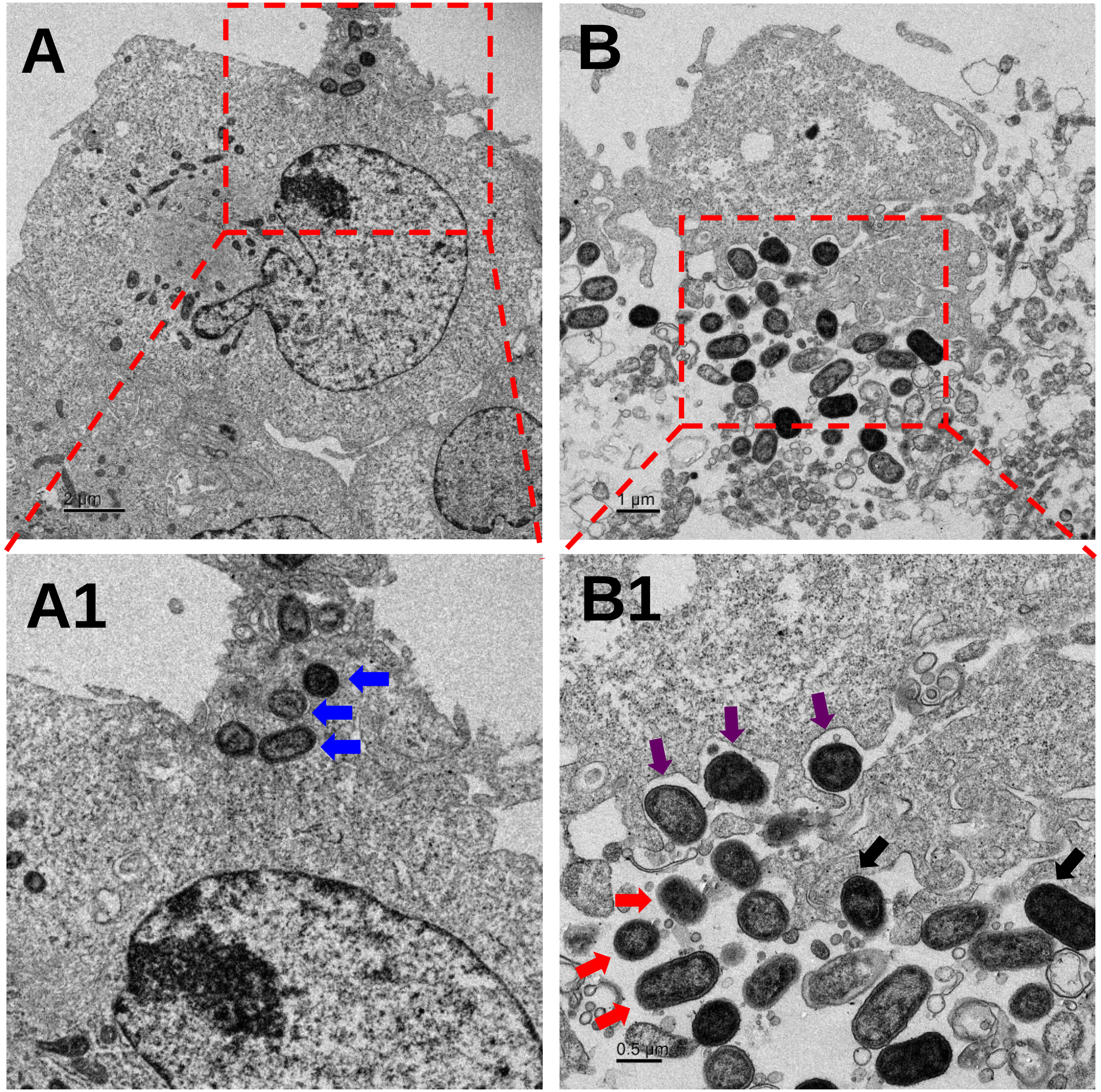
A
2 µm
B
1 µm
A1
B1
0.5 µm

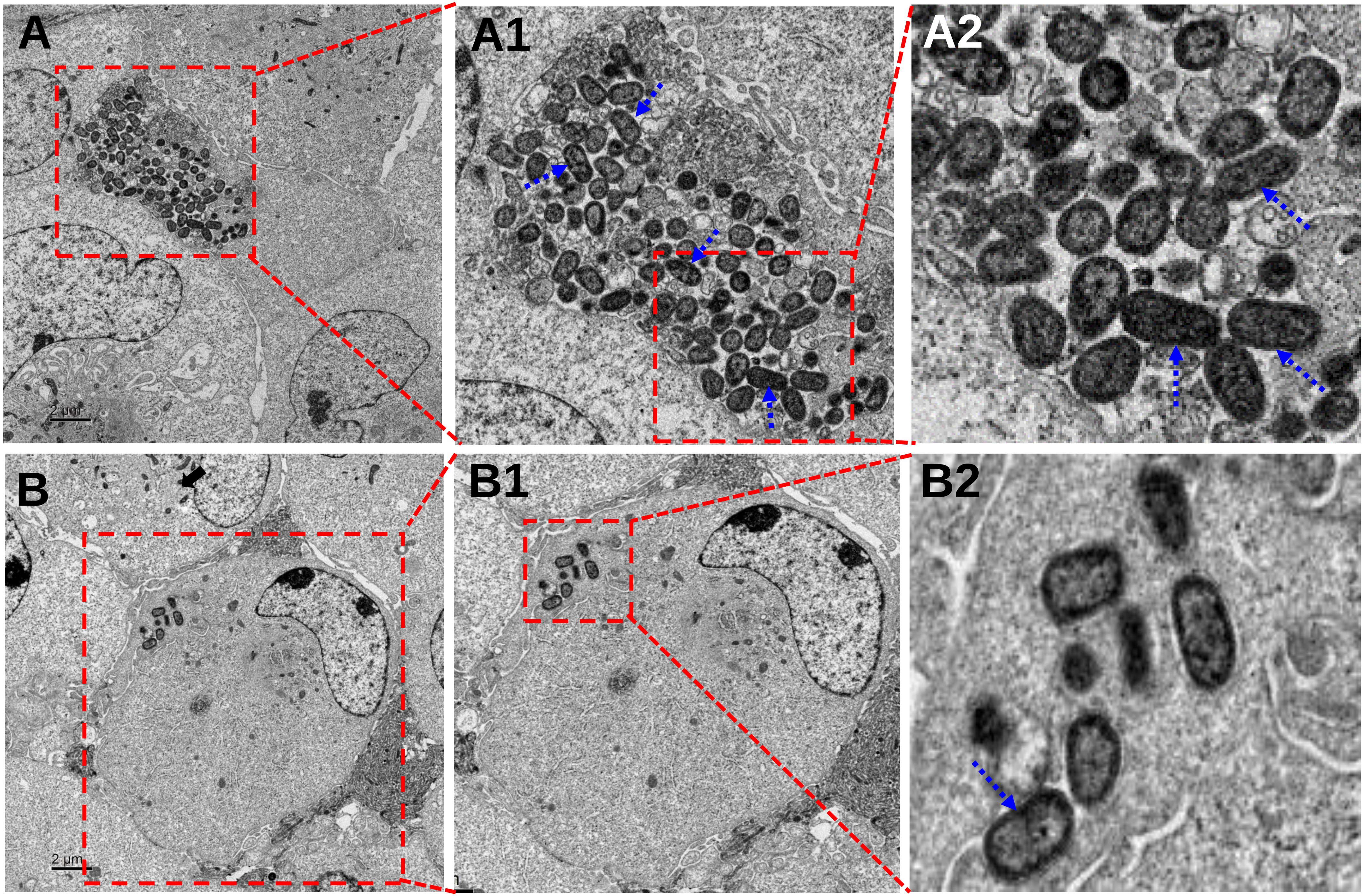
A
A1
A2
2 μm
B
B1
B2
2 μm

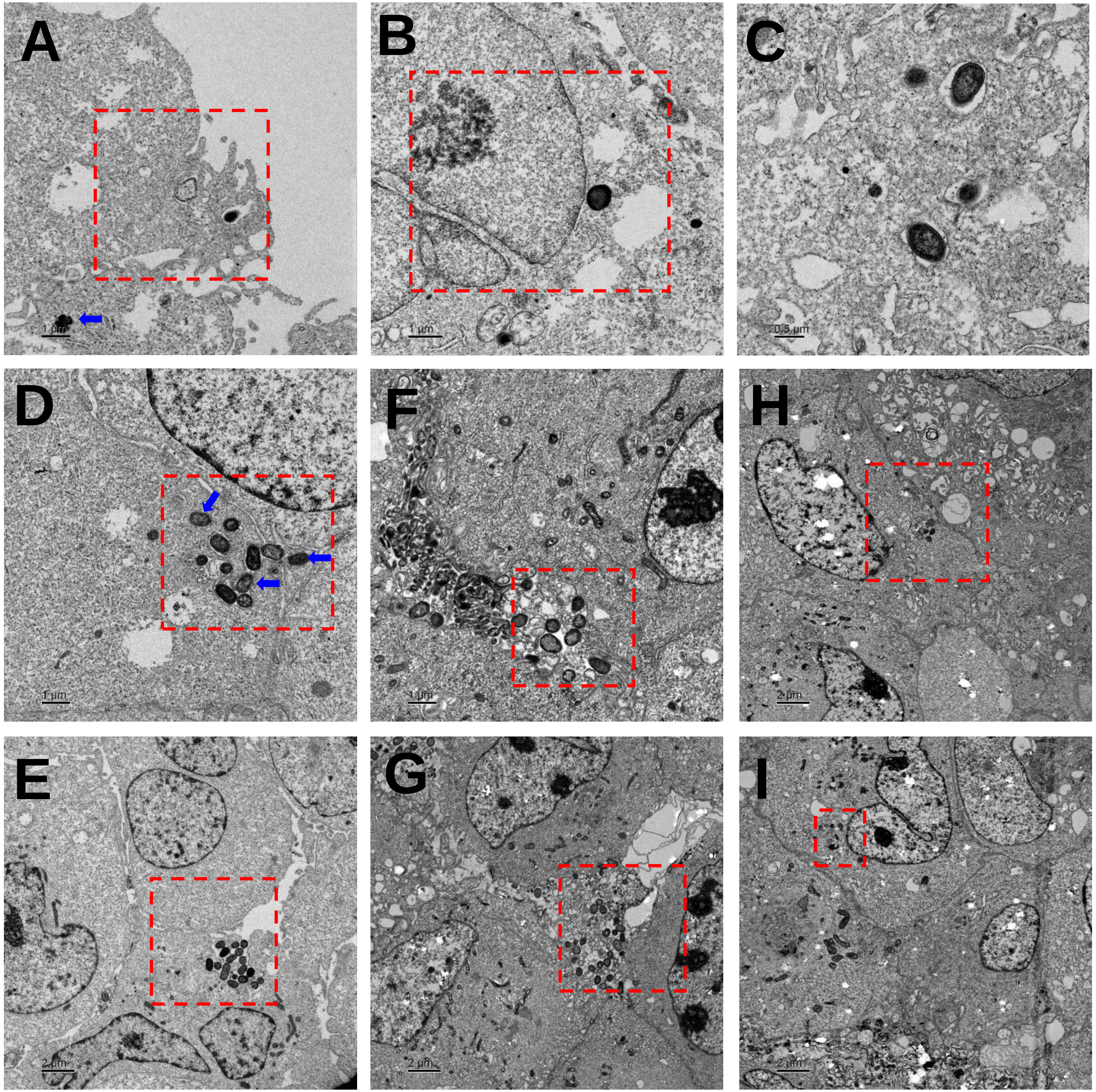

A
1 µm
B
1 µm
C
0.5 µm
D
1 µm
F
1 µm
H
2 µm
E
2 µm
G
2 µm
I
2 µm

Schematic representation for adhesion and intracellular survival of *Pg* strains in ARPE-19 cells

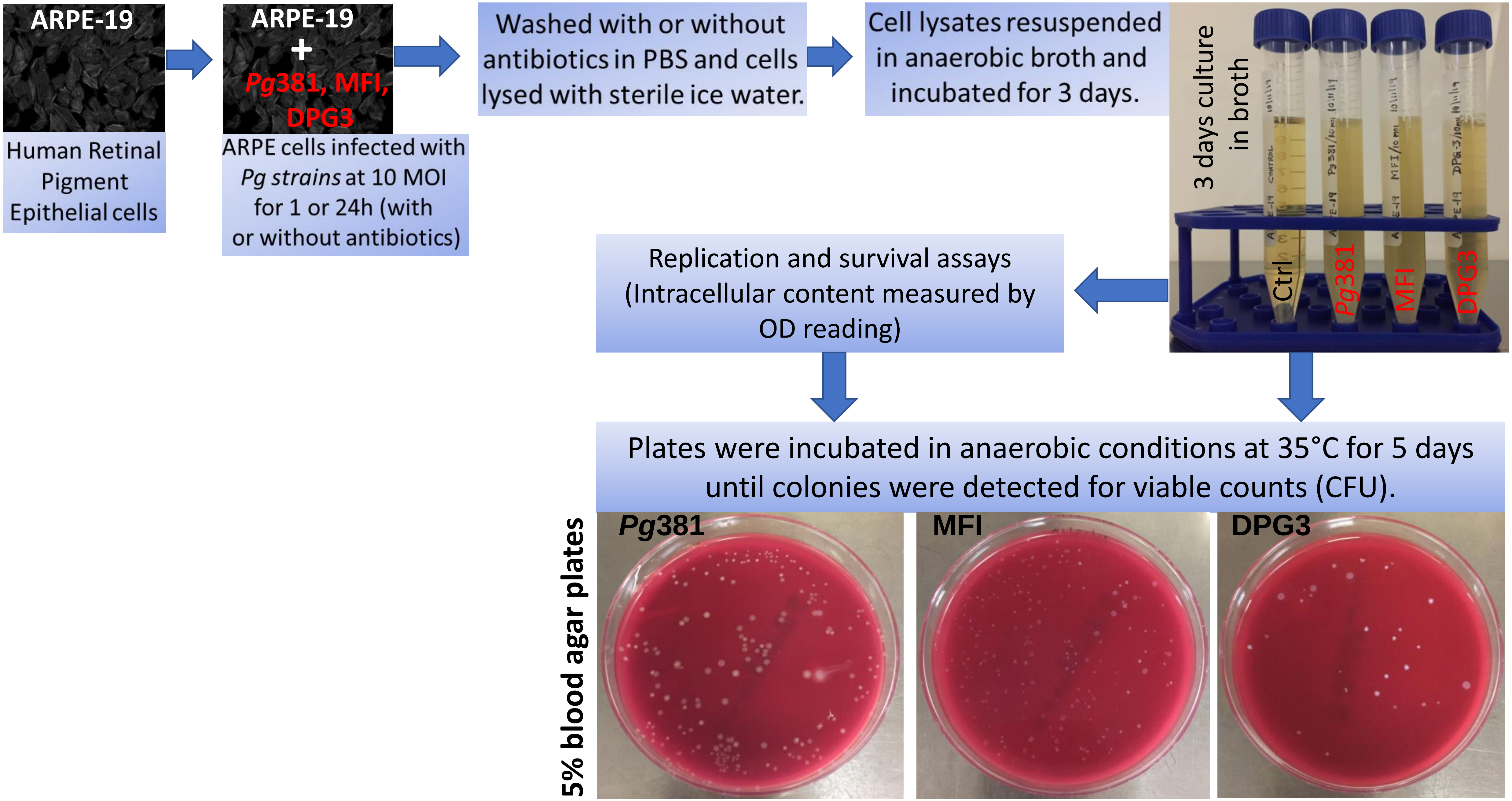

Supplement: Supplementary file 1 — Supplementary information. [file 41598_2020_64449_MOESM1_ESM.pdf]
